# Phase I/II study of the LAG-3 inhibitor ieramilimab (LAG525) ± anti-PD-1 spartalizumab (PDR001) in patients with advanced malignancies

**DOI:** 10.1136/jitc-2021-003776

**Published:** 2022-02-25

**Authors:** Patrick Schöffski, Daniel S W Tan, Miguel Martín, María Ochoa-de-Olza, John Sarantopoulos, Richard D Carvajal, Chrisann Kyi, Taito Esaki, Amy Prawira, Wallace Akerley, Filippo De Braud, Rina Hui, Tian Zhang, Ross A Soo, Michela Maur, Andrew Weickhardt, Jürgen Krauss, Barbara Deschler-Baier, Allen Lau, Tanay S Samant, Tyler Longmire, Niladri Roy Chowdhury, Catherine A Sabatos-Peyton, Nidhi Patel, Radha Ramesh, Tiancen Hu, Ana Carion, Daniel Gusenleitner, Padmaja Yerramilli-Rao, Vasileios Askoxylakis, Eunice L Kwak, David S Hong

**Affiliations:** 1 Department of General Medical Oncology, Leuven Cancer Institute, University Hospitals Leuven, Leuven, Belgium; 2 National Cancer Centre Singapore, Singapore; 3 Duke-NUS Medical School, Singapore; 4 Hospital General Universitario Gregorio Maranon, Madrid, Spain; 5 Vall d'Hebron University Hospital, Barcelona, Spain; 6 Institute for Drug Development, Mays Cancer Center at University of Texas Health San Antonio MD Anderson Cancer Center, San Antonio, Texas, USA; 7 Columbia University Irving Medical Center, New York, New York, USA; 8 Memorial Sloan Kettering Cancer Center, New York, New York, USA; 9 National Hospital Organization Kyushu Cancer Center, Fukuoka, Japan; 10 Princess Margaret Hospital Cancer Centre, Toronto, Ontario, Canada; 11 Huntsman Cancer Institute, University of Utah, Salt Lake City, Utah, USA; 12 Fondazione IRCCS, Istituto Nazionale dei Tumori, Milan, Italy; 13 Westmead Hospital and The University of Sydney, Sydney, New South Wales, Australia; 14 University of Texas Southwestern Medical Center, Dallas, Texas, USA; 15 National University Cancer Institute, Singapore; 16 Oncologia Medica, AOU Policlinico di Modena, Modena, Emilia-Romagna, Italy; 17 Austin Health, Heidelberg, Victoria, Australia; 18 National Center for Tumor Diseases, Heidelberg, Germany; 19 Universitätsklinikum Würzburg, Wurzburg, Germany; 20 Novartis Institutes for BioMedical Research Inc, Cambridge, Massachusetts, USA; 21 The University of Texas MD Anderson Cancer Center, Houston, Texas, USA

**Keywords:** immunotherapy, drug therapy, combination

## Abstract

**Background:**

Lymphocyte-activation gene 3 (LAG-3) is an inhibitory immunoreceptor that negatively regulates T-cell activation. This paper presents preclinical characterization of the LAG-3 inhibitor, ieramilimab (LAG525), and phase I data for the treatment of patients with advanced/metastatic solid tumors with ieramilimab ±the anti-programmed cell death-1 antibody, spartalizumab.

**Methods:**

Eligible patients had advanced/metastatic solid tumors and progressed after, or were unsuitable for, standard-of-care therapy, including checkpoint inhibitors in some cases. Patients received ieramilimab ±spartalizumab across various dose-escalation schedules. The primary objective was to assess the maximum tolerated dose (MTD) or recommended phase II dose (RP2D).

**Results:**

In total, 255 patients were allocated to single-agent ieramilimab (n=134) and combination (n=121) treatment arms. The majority (98%) had received prior antineoplastic therapy (median, 3). Four patients experienced dose-limiting toxicities in each treatment arm across various dosing cohorts. No MTD was reached. The RP2D on a 3-week schedule was declared as 400 mg ieramilimab plus 300 mg spartalizumab and, on a 4-week schedule (once every 4 weeks; Q4W), as 800 mg ieramilimab plus 400 mg spartalizumab; tumor target (LAG-3) suppression with 600 mg ieramilimab Q4W was predicted to be similar to the Q4W, RP2D schedule. Treatment-related adverse events (TRAEs) occurred in 75 (56%) and 84 (69%) patients in the single-agent and combination arms, respectively. Most common TRAEs were fatigue, gastrointestinal, and skin disorders, and were of mild severity; seven patients experienced at least one treatment-related serious adverse event in the single-agent (5%) and combination group (5.8%). Antitumor activity was observed in the combination arm, with 3 (2%) complete responses and 10 (8%) partial responses in a mixed population of tumor types. In the combination arm, eight patients (6.6%) experienced stable disease for 6 months or longer versus six patients (4.5%) in the single-agent arm. Responding patients trended towards having higher levels of immune gene expression, including *CD8* and *LAG3*, in tumor tissue at baseline.

**Conclusions:**

Ieramilimab was well tolerated as monotherapy and in combination with spartalizumab. The toxicity profile of ieramilimab in combination with spartalizumab was comparable to that of spartalizumab alone. Modest antitumor activity was seen with combination treatment.

**Trial registration number:**

NCT02460224.

## Introduction

Lymphocyte-activation gene 3 (LAG-3) is an inhibitory immunoreceptor expressed on immune cells including activated T cells,^1^ T-regulatory cells,^2^ natural killer (NK) cells,^1^ plasmacytoid dendritic cells,^3^ and natural regulatory plasma cells.^4^ LAG-3 associates with cluster of differentiation (CD)3 in the T-cell receptor complex and negatively regulates signal transduction, leading to reduced T-cell proliferation and cytokine production.^5^ LAG-3 has high affinity for its best-characterized ligand, major histocompatibility complex class II (MHC-II)^1^; other described ligands include galectin-3,^6^ L-SECtin,^7^ and fibrinogen-like protein 1 (FGL-1).^8^ Interaction between LAG-3 and its ligands results in inhibition of T-cell activation.^1 6–8^

Sustained T-cell activation within a chronic inflammatory environment, including tumors, increases LAG-3 co-expression with co-inhibitory receptors, including programmed cell death-1 (PD-1).^1 9^ Sustained expression of these immune cell checkpoints can alter immune responses and contribute to T-cell suppression and subsequent immune dysfunction.^1 9^ Dysregulation of immune checkpoints is a key mechanism by which tumors evade immune surveillance.^9^ Blockade of LAG-3 has been shown to improve cytotoxic T-lymphocyte proliferation and effector function in vivo.^10 11^ In addition, independent of MHC-II, LAG-3 has been shown to associate with the liver-secreted protein, FGL-1.^8^ Blockade of the FGL-1–LAG-3 interaction by monoclonal antibodies (mAbs) suppressed tumor growth in established mouse models, in a receptor–ligand interdependent manner.^8^

Data from syngeneic mouse models demonstrated that dual LAG-3/PD-1 blockade reduced tumor growth by increasing the proportion of effector T cells in the tumor.^12^ A number of LAG-3–targeting molecules are currently in early stages of clinical development, with early results suggesting a modest benefit of single-agent, anti-LAG-3 treatment, supporting the potential of combination approaches.^13^

Ieramilimab (LAG525) is a humanized immunoglobulin 4 (IgG4) (S228P) mAb that binds to LAG-3, resulting in inhibition of LAG-3 interaction with MHC-II molecules. Spartalizumab is a humanized IgG4 anti-PD-1 (S228P) mAb, which binds to PD-1 and blocks the interaction between the receptor and its ligands, programmed death-ligand 1 (PD-L1), and programmed death-ligand 2 (PD-L2).^14^ Spartalizumab has shown clinical efficacy in various malignancies, including non-small cell lung cancer (NSCLC),^15^ melanoma,^15^ anaplastic thyroid cancer,^16^ neuroendocrine neoplasms,^17^ and nasopharyngeal cancer.^18^

In this report, we present the preclinical characterization of ieramilimab and clinical data from a phase I study investigating ieramilimab as both a single agent and in combination with spartalizumab for the treatment of patients with advanced/metastatic solid tumors.

## Methods

### Preclinical characterization of ieramilimab

Ieramilimab is a humanized IgG4 antibody that contains the S228 hinge-stabilizing mutation and blocks the LAG-3–MHC-II interaction with low nanomolar affinity (data not shown). A plate-based Meso Scale Discovery (MSD) assay was developed to determine the ability of ieramilimab to neutralize the interaction between plate-bound FGL-1–His protein and biotinylated LAG-3–Fc protein. To establish the role of ieramilimab in enhancing cytokine secretion, naive B cells and T follicular helper (Tfh) cells were isolated from healthy human donor peripheral blood mononuclear cells and activated with Staphylococcal enterotoxin B (SEB) in the presence of ieramilimab or human IgG4 isotype control; supernatants were harvested, and cytokines were measured by MSD. The crystal structure of a human LAG-3 (first immunoglobulin variable domain (D1)) bound to the antigen-binding fragment of a humanized anti-LAG-3 antibody, ieramilimab, was determined. Detailed preclinical methods for in vitro assays and X-ray crystallography can be found in the online supplemental file (online only).

10.1136/jitc-2021-003776.supp1 Supplementary data

### Study oversight

This study was performed in accordance with the Declaration of Helsinki and the principles of Good Clinical Practice and was approved by an independent ethics committee or Institutional Review Board at each study center. All patients provided written informed consent before any study procedures. The study was sponsored by Novartis Pharmaceuticals Corporation, which provided the study drug and worked with the investigators to design the study, collect, analyze, and interpret data.

### Clinical study design

This phase I/II, open-label, multicenter study investigated the safety and efficacy of single-agent ieramilimab and in combination with spartalizumab in patients with advanced solid malignancies. Phase I consisted of two, staggered, dose-escalation arms: single-agent ieramilimab followed by ieramilimab in combination with spartalizumab.

Following completion of phase I, phase II was conducted in selected cancer indications.

Here, we present the data from phase I; data cut-off June 1, 2020.

### Study objectives

The primary objective of phase I was to estimate the recommended phase II dose (RP2D) or maximum tolerated dose (MTD) of both single-agent ieramilimab and ieramilimab in combination with spartalizumab. Key secondary objectives included characterization of the safety and tolerability of single-agent ieramilimab and ieramilimab in combination with spartalizumab, assessment of pharmacokinetics (PK), and evaluation of preliminary antitumor activity. Biomarker analysis of pharmacodynamic effects was a key exploratory objective.

### Patient population

Eligible patients for phase I were adults (≥18 years) with advanced/metastatic solid tumors who had either progressed on, were intolerant to, or were unsuitable for standard therapy, with an Eastern Cooperative Oncology Group (ECOG) performance status ≤2. Where feasible, patients were required to provide a new tumor biopsy at baseline and during treatment.

Key exclusion criteria were presence of symptomatic central nervous system (CNS) metastases or CNS metastases requiring local surgery; clinically significant cardiac disease or impairment; autoimmune disease; history of, or current, drug-induced pneumonitis; and systemic treatment with immunosuppressive medication, which could interfere with the study drugs, other than replacement-dose corticosteroids in the setting of adrenal insufficiency.

### Drug administration

Ieramilimab and spartalizumab were administered separately via intravenous infusions over 30 min, with at least a 30-min break between administration of the two antibodies. Infusions for each antibody could be extended to up to 2 hours if clinically indicated, and the break between ieramilimab and spartalizumab infusions could be extended to up to 4 hours if clinically indicated. Ieramilimab was given first, followed by spartalizumab.

### Treatment plan

The ieramilimab and spartalizumab starting doses were both 1 mg/kg, administered via intravenous infusion once every 2 weeks (Q2W). The starting doses were determined from toxicology studies and efficacy data of comparable checkpoint inhibitors. Initially, ieramilimab was administered Q2W, consistent with a schedule commonly used for other mAbs with a similar PK profile. In the single-agent arm, patients received ieramilimab Q2W (1 mg/kg, 3 mg/kg, 5 mg/kg, 10 mg/kg, 15 mg/kg, 240 mg, 400 mg) or once every 4 weeks (Q4W; 3 mg/kg, 5 mg/kg, 10 mg/kg, 400 mg). In the combination arm, patients received ieramilimab and spartalizumab Q2W (0.3 mg/kg/1 mg/kg, 1 mg/kg/1 mg/kg, 80 mg/80 mg, 80 mg/240 mg, 240 mg/240 mg), once every 3 weeks (Q3W; 240 mg/300 mg, 400 mg/300 mg, 600 mg/300 mg) or Q4W (80 mg/240 mg, 400 mg/400 mg, 800 mg/400 mg, 1000 mg/400 mg), or ieramilimab Q2W and spartalizumab Q4W (80 mg/400 mg, 240 mg/400 mg, 300 mg/400 mg). One cycle was defined as 28 days for patients on a Q4W schedule and 21 days for patients on a Q3W schedule.

Treatment continued until unacceptable toxicity, progressive disease (PD) as per immune-related response criteria (irRC),^19^ or patient/physician decision; guidelines are provided in the online supplemental file (online only). Treatment was also discontinued if consecutive doses (≥2) were missed due to drug-related toxicities; study treatment could be continued beyond disease progression for clinical benefit.

Dose-escalation decisions were based on all available safety, dose-limiting toxicity (DLT), PK, and pharmacodynamic data, and were guided by a Bayesian hierarchical logistic regression model following the escalation with overdose control principle. Dose escalation occurred until the MTD or RP2D was determined.

### Safety assessments

Safety assessments included incidence and severity of adverse events (AEs) and serious AEs (SAEs), changes in laboratory values, physical examination, vital signs, ECOG performance status, and cardiac assessments. AEs were defined by the National Cancer Institute Common Terminology Criteria for Adverse Events V.4.03 and assessed at every visit. A DLT was defined as an AE of grade ≥3, suspected to be related to the study drug. The window for DLTs was one cycle for single-agent ieramilimab (eg, 28 days for Q4W and Q2W) and two cycles for ieramilimab and spartalizumab combination (eg, 56 days for a Q4W schedule and 42 days for a Q3W schedule).

### Response assessments

Efficacy was evaluated by local investigator assessment per Response Evaluation Criteria In Solid Tumors (RECIST) V.1.1 and irRC. Tumor assessments were performed at screening (maximum 21 days before start of treatment); every 8 weeks (±1 week) after cycle 1, day 1 until 40 weeks, and then every 12 weeks (±1 week) until disease progression per irRC, or withdrawal from the study.

### Assessment of PK

Blood samples for PK assessments were collected on days 1, 2, 8, 11, and 15 in cycles 1 and 3; day 1 in cycles 2, 4, 5, and 6; and at the end of treatment. Serum concentrations were determined with liquid chromatography mass spectrometry.

### Biomarker assessments

Biopsy samples were collected at screening/baseline and between cycle 3 days 1–15; some on-treatment samples were provided during cycle 2, prior to a protocol amendment aligning samples with preclinical evidence on the timing of immune response to PD-1 blockade. Archival tumor samples were used for biomarker assessments in a limited number of cases.

For baseline and on-treatment samples, immune marker expression was assessed by immunohistochemistry (IHC) and gene expression by RNA-based analysis (further details can be found in the online supplemental file, online only).

### Statistical methods

To declare the MTD, the following thresholds needed to be met: at least 6 patients treated at a given dose and a minimum of 21 patients for the single-agent arm of the trial or 15 patients for the combination arm. This given dose was recommended following review of all clinical data by Novartis and investigators.

Preclinical methodology is described in the online supplemental material (online only).

## Results

### Preclinical characterization of ieramilimab

**Figure 1:**
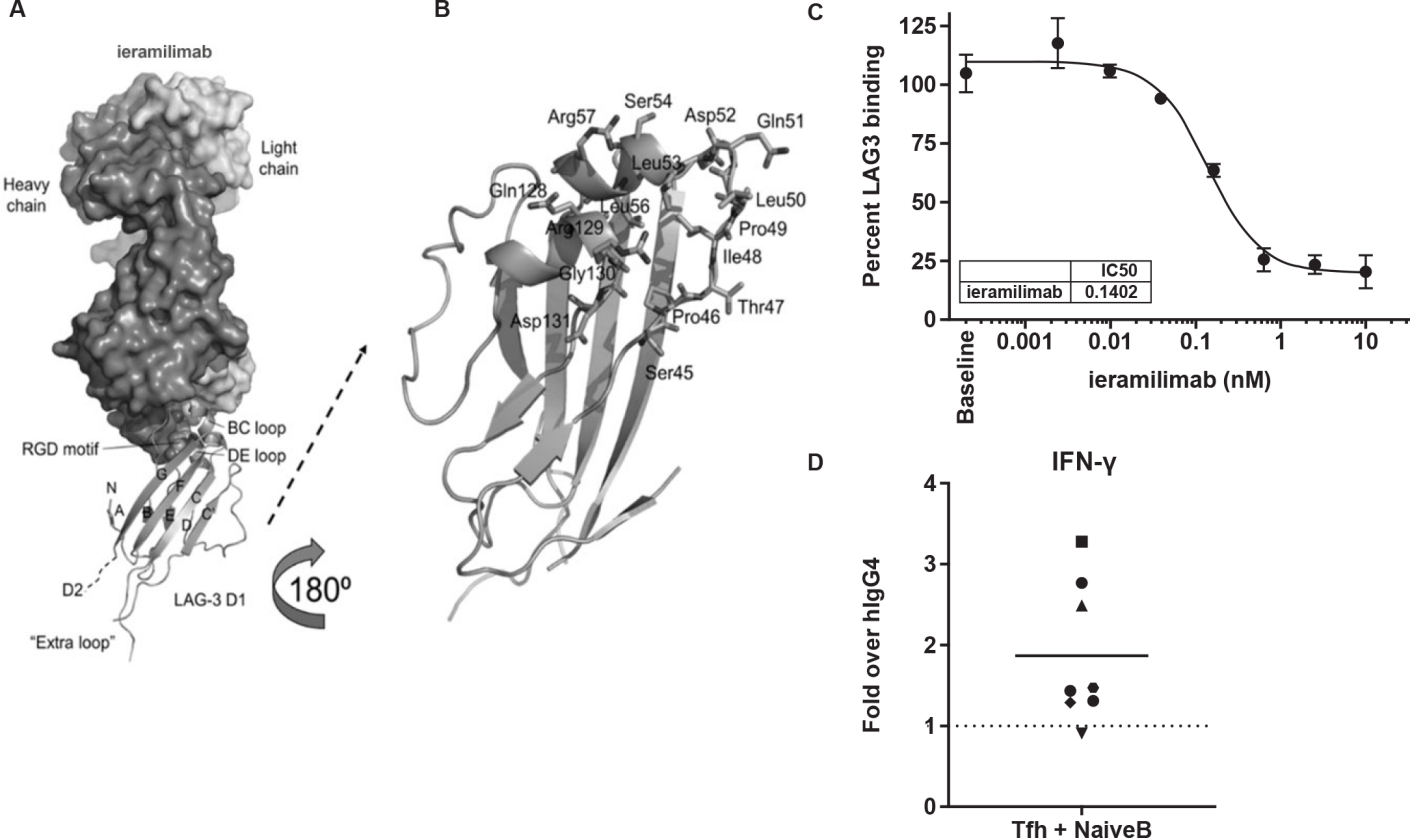
Preclinical characterization of ieramilimab. (A) Overall structure of ieramilimab antigen-binding fragment binding to LAG-3. Shown are (i) the heavy and light chains of ieramilimab in surface and LAG-3 domain 1 (D1) in ribbons, (ii) the N-terminus (N), the names of the β stands, and the C-terminus of D1 that leads to domain 2 (D2) of LAG-3, (iii) the BC and DE loops of LAG-3 that comprise the epitope of ieramilimab, in which the RGD motif critical for binding MHC-II is shown as spheres, and (iv) the unique ‘extra loop’ of LAG-3, which is far away from the ieramilimab epitope. (B) Detailed view of ieramilimab epitope residues on LAG-3 (shown as sticks and labeled). (C) Ieramilimab blocks the binding of FGL-1 to LAG-3. (D) In three out of eight healthy human donors assayed, ieramilimab enhances IFN-γ secretion in Tfh/B cell co-cultures stimulated with SEB, relative to hIgG4 isotype control. FGL-1, fibrinogen-like protein 1; hIgG4, human immunoglobulin G4; IFN, interferon; LAG-3, lymphocyte-activation gene 3; MHC-II, major histocompatibility complex class II; RGD, arginylglycylaspartic acid; SEB, Staphylococcal enterotoxin B; Tfh, T follicular helper.

Ieramilimab demonstrated binding to D1 of LAG-3 through several continuous and discontinuous sequences covering the BC and DE loops, as well as the arginylglycylaspartic acid motif (figure 1A, B). The recently described FGL-1–LAG-3 interaction has been reported to occur within D1 and D2 of LAG-3, independent of the MHC-II–LAG-3 interaction.^8^ Using a novel MSD assay, we determined that ieramilimab blocked the LAG-3–FGL-1 interaction with a half-maximal inhibitory concentration (IC_50_) of approximately 0.1 nM (figure 1C). In three out of eight healthy donors tested, in a co-culture of SEB-stimulated Tfh cells and B cells (online supplemental methods, online only), interferon gamma (IFN-γ) secretion was increased by blockade of LAG-3 with ieramilimab, relative to IgG control (figure 1D), demonstrating a functional ability of ieramilimab to enhance a T-cell response.

### Patient demographics/characteristics

As of the data cut-off, June 1, 2020, 255 patients were treated in phase I: 134 patients received single-agent ieramilimab, either Q2W (n=107) or Q4W (n=27), and 121 patients received combination ieramilimab and spartalizumab, either Q2W (n=29), Q3W (n=38), or Q4W (n=31), or ieramilimab Q2W plus spartalizumab Q4W (n=23).

Patient demographics and baseline characteristics are shown in table 1. The median age was 59 years (range 26–81) and 58 years (range 19–77) for the single-agent and combination groups, respectively. Overall, 249 (98%) patients had received prior antineoplastic therapies, with a median of three prior therapies, including prior checkpoint inhibitor therapy in some cases. In the single-agent treatment group, 133 patients (99.3%) discontinued treatment due to PD (n=117, 87.3%), patient/guardian decision (n=9, 6.7%), death (n=4, 3%), physician decision (n=2, 1.5%), and AE incidence (n=1, 0.7%). One patient with renal cell carcinoma who received ieramilimab monotherapy with shrinkage of target lesions switched to combination treatment due to worsening, non-measurable disease. In the combination treatment group, 119 (98.3%) patients discontinued treatment due to PD (n=89, 73.6%), physician decision (n=10, 8.3%), death (n=8, 6.6%), AEs (n=6, 5%), and patient/guardian decision (n=5, 4.1%), with one (0.8%) lost to follow-up.

**Table 1:** Patient demographics

Demographic variable	All phase I SA patients (N=134)	All phase I combo patients (N=121)	All phase I patients (N=255)
Age, years			
Median	59.0	58.0	58.0
Minimum–maximum	26–81	19–77	19–81
Sex, n (%)			
Male	65 (48.5)	55 (45.5)	120 (47.1)
Female	69 (51.5)	66 (54.5)	135 (52.9)
ECOG performance status, n (%)			
0	51 (38.1)	45 (37.2)	96 (37.6)
1	78 (58.2)	73 (60.3)	151 (59.2)
2	4 (3.0)	3 (2.5)	7 (2.7)
Missing	1 (0.7)	0 (0)	1 (0.4)
Prior antineoplastic therapies, n			
Median	3.0	3.0	–
Minimum–maximum	1–11	1–14	–
Checkpoint inhibitors (Anti-CTLA-4, PD-1, or PD-L1), n (%)	51 (38.1)	22 (18.2)	73 (28.6)
Tumor type (≥2%), n (%)			
Non-small cell lung cancer	20 (14.9)	8 (6.6)	28 (11)
Colorectal cancer	14 (10.4)	7 (5.8)	21 (8.2)
Cutaneous melanoma	13 (9.7)	5 (4.1)	18 (7.1)
Metastatic renal cell carcinoma	7 (5.2)	4 (3.3)	11 (4.3)
Sarcoma	2 (1.5)	12 (9.9)	14 (5.5)
Ovarian cancer	7 (5.2)	7 (5.8)	14 (5.5)
Mesothelioma	2 (1.5)	8 (6.6)	10 (3.9)
Bladder cancer	1 (0.7)	6 (5.0)	7 (2.7)
Hepatocellular carcinoma	7 (5.2)	0	7 (2.7)
Pancreatic cancer	1 (0.7)	6 (5.0)	7 (2.7)
Breast cancer	5 (3.7)	3 (2.5)	8 (3.1)
Cervical cancer	3 (2.2)	2 (1.7)	5 (2)
Endometrial cancer	6 (4.5)	4 (3.3)	10 (3.9)
Malignant neoplasm of thymus	3 (2.2)	4 (3.3)	7 (2.8)
Nasopharyngeal cancer	3 (2.2)	5 (4.1)	8 (3.1)
Neuroendocrine	3 (2.2)	2 (1.7)	5 (2)
Non-cutaneous melanoma	3 (2.2)	2 (1.7)	5 (2)
Prostate cancer	5 (3.7)	1 (0.8)	6 (2.4)
Head and neck cancer	4 (3.0)	5 (4.1)	9 (3.5)
Triple-negative breast cancer	0	5 (4.1)	5 (2)
Other*	28 (20.9)	25 (20.7)	53 (20.8)

*Other indications included: Basal cell carcinoma, cholangiocarcinoma, esophageal cancer, gallbladder cancer, gastric cancer, gastrointestinal stromal tumor, liposarcoma, small cell lung cancer, small intestine cancer, testicular cancer, and uveal melanoma.

CTLA-4, cytotoxic T-lymphocyte-associated protein 4; ECOG, Eastern Cooperative Oncology Group; PD-1, programmed cell death-1; PD-L1, programmed death-ligand 1; SA, single-agent.

### Safety

AEs, regardless of study drug relationship, were observed in 132 (98.5%) and 120 (99.2%) patients in the single-agent and combination groups, respectively, and were comparable between treatment arms.

The most common (≥20%) AEs experienced by the single-agent group were fatigue (n=36, 26.9%), nausea (n=35, 26.1%), anemia (n=33, 24.6%), constipation (n=33, 24.6%), decreased appetite (n=33, 24.6%), abdominal pain (n=30, 22.4%), dyspnea (n=30, 22.4%), and vomiting (n=27, 20.1%). The most common (≥20%) AEs experienced by the combination group were fatigue (n=44, 36.4%), nausea (n=44, 36.4%), diarrhea (n=36, 29.8%), decreased appetite (n=36, 29.8%), constipation (n=29, 24.0%), vomiting (n=28, 23.1%), cough (n=28, 23.1%), dyspnea (n=26, 21.5%), and anemia (n=26, 21.5%).

Grade 3/4 AEs were observed in 75 (56.0%) and 66 (54.5%) patients in the single-agent and combination groups, respectively.

**Figure 2:**
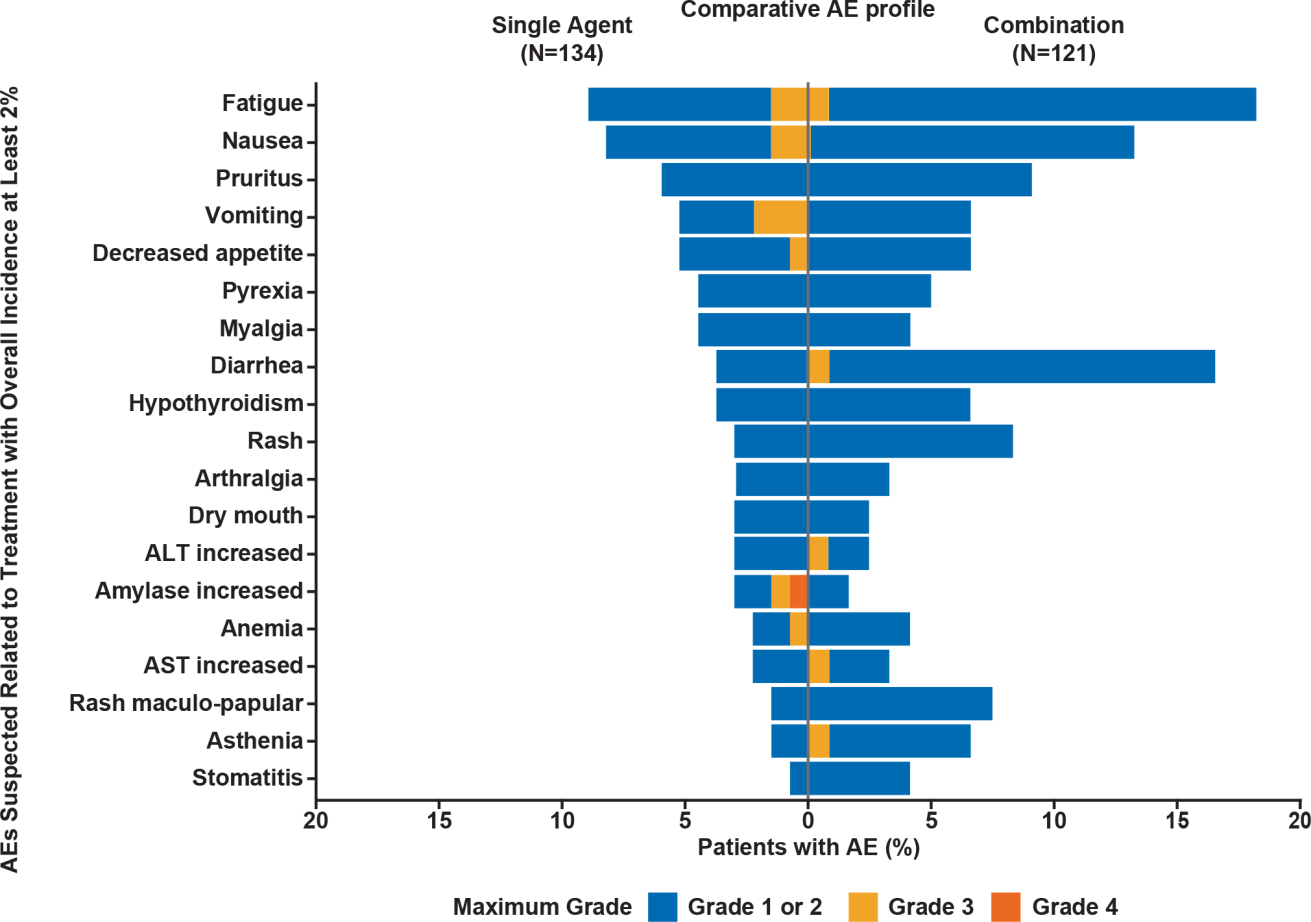
AEs per CTCAE V.4.03, suspected to be treatment related, with an overall incidence of at least 2% for both the single-agent ieramilimab arm and the spartalizumab combination arm. AE, adverse event; ALT, alanine aminotransferase; AST, aspartate aminotransferase; CTCAE, Common Terminology Criteria for Adverse Events.

Treatment-related AEs (TRAEs) of any grade were reported in 75 (56.0%) and 84 (69.4%) patients in the single-agent and combination groups, respectively. TRAEs of grade 3/4 were experienced by 9 (6.7%) patients in the single-agent group and 11 (9.1%) patients in the combination group (figure 2; online supplemental table A1, online only). The most common (≥10 patients) TRAEs of any grade were fatigue (n=12, 9.0%) and nausea (n=11, 8.2%) in the single-agent group. Low grade, treatment-related changes in thyroid function were reported in some patients (<5%); however, no other endocrinopathies or immune-related AEs were reported in the single-agent group. In the combination group, the most common TRAEs of any grade were fatigue (n=22, 18.2%), diarrhea (n=20, 16.5%), nausea (n=15, 12.4%), pruritus (n=12, 9.9%), and rash (n=10, 8.3%). The most common grade 3/4 TRAEs (≥3 patients) in all patients in the phase I study included lipase increase (n=4; 1.6%), fatigue (n=3; 1.2%), and vomiting (n=3; 1.2%; online supplemental table A1). Immune-related TRAEs reported in the combination group included colitis, hepatitis, polyarthritis, and hyperglycemia (diabetic ketoacidosis).

Five (4.1%) patients discontinued treatment due to TRAEs in the combination arm; the TRAEs were immune-related colitis and diarrhea, brain tumor edema, pneumonitis, blurred vision, fatigue, and autoimmune hepatitis. In addition, treatment was discontinued for one patient in the combination arm due to grade 3 abdominal pain associated with clinical progression. No TRAEs led to treatment discontinuation in the single-agent arm.

SAEs, regardless of study drug relationship and of any grade, were reported in 52 (38.8%) patients and 59 (48.8%) patients in the single-agent and combination groups, respectively. In the single-agent group, seven (5.2%) patients experienced at least one treatment-related SAE (TRSAE); the most common (≥1 patient) TRSAEs were vomiting (n=3, 2.2%) and diarrhea (n=2, 1.5%). Six (4.5%) patients had a fatal SAE, one (0.7%) of which, acute kidney injury, was considered treatment related; this patient experienced acute kidney injury secondary to worsening extensive tumor burden with histologic tumor necrosis consistent with grade 4 tumor lysis syndrome. In the combination group, seven (5.8%) patients experienced at least one TRSAE. Seven (5.8%) patients experienced a fatal SAE, none of which were treatment related.

### DLTs

Four (3.0%) patients experienced at least one DLT in the single-agent ieramilimab treatment group: one patient (0.7%) grade 4 acute kidney injury (ieramilimab 10 mg/kg Q4W), one patient (0.7%) grade 3 intra-abdominal fluid collection (ieramilimab 1 mg/kg Q2W), one patient (0.7%) grade 3 lipase increase (ieramilimab 5 mg/kg Q2W), and one patient (0.7%) grade 3 vomiting (ieramilimab 5 mg/kg Q2W).

In the combination group, four (3.3%) patients experienced at least one DLT: one patient (0.8%) grade 4 autoimmune hepatitis and grade 3 fatigue (ieramilimab 1000 mg Q4W+spartalizumab 400 mg Q4W), one patient (0.8%) grade 3 hyperglycemia (ieramilimab 80 mg Q2W+spartalizumab 400 mg Q4W), one patient (0.8%) grade 3 brain tumor edema (ieramilimab 600 mg Q3W+spartalizumab 300 mg Q3W), and one patient (0.8%) grade 3 pneumonitis (ieramilimab 400 mg Q4W+spartalizumab 400 mg Q4W). A MTD was not reached, similar to other phase I trials of checkpoint inhibitors.^20^ Therefore, the RP2D was determined using a population PK/pharmacodynamic modeling approach, coupled with a prediction for intratumor receptor occupancy,^21^ to estimate 90% target engagement in >90% of patients. The RP2D on a Q3W schedule was 400 mg ieramilimab in combination with 300 mg spartalizumab and, on a Q4W schedule, the RP2D was 800 mg ieramilimab in combination with 400 mg spartalizumab. Also, the population PK/ pharmacodynamic analysis predicted that tumor target (LAG-3) suppression at 600 mg ieramilimab Q4W is similar to the Q4W RP2D schedule (data on file).

### PK of ieramilimab as single agent and ieramilimab in combination with spartalizumab

**Figure 3:**
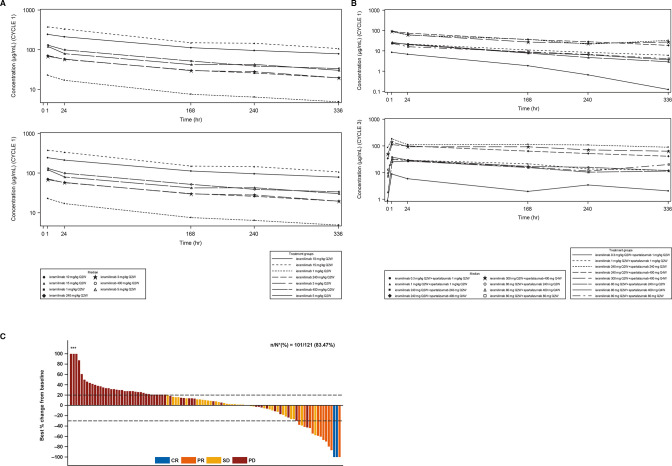
Pharmacokinetics and best percentage change in tumors. (A) Median concentration–time profiles for Q2W dosing regimens for SA ieramilimab. (B) Median concentration–time profiles for Q2W dosing regimens for ieramilimab in combination with spartalizumab. (C) Waterfall plot for best percentage change of predefined target lesions from baseline in sum of longest diameters based on local radiology review of RECIST V.1.1 for patients treated with ieramilimab +spartalizumab *Indicates the bars where best percentage change from baseline has been cut at 100%. CR, complete response; PD, progressive disease; PR, partial response; Q2W, every 2 weeks; Q4W, every 4 weeks; RECIST, Response Evaluation Criteria In Solid Tumors; SA, single agent; SD, stable disease.

For both treatment groups, following ieramilimab treatment infusion, approximately dose-proportional increases in ieramilimab exposure (cycle 1 area under the plasma concentration–time curve (AUC_tau_)) were observed from 1 mg/kg to 15 mg/kg, as suggested by an approximate 20-fold increase in exposure with a 15-fold increase in dose (figure 3A, B; online supplemental table A2; online only).

Based on single-agent and combination dosing regimen data (Q2W, Q3W, and Q4W), exposure (eg, maximum concentration (C_max_) or AUC_tau_) during cycle 3 was higher compared with cycle 1, indicating moderate accumulation of ieramilimab. PK variability was low-to-moderate, as illustrated by between-subject variability (CV%), including a C_max_ of 13.8%–34.6% and an AUC_tau_ of 17.3%–45.6% at cycle 1 day 1 (N>3). The observed median effective half-life accounting for drug accumulation (T_1/2, eff_) of ieramilimab at cycle 3 ranged from 10 to 23 days.

The PK of ieramilimab in combination with various doses of spartalizumab were comparable to those of single-agent ieramilimab at the same dose levels. At cycle 1, exposure of 240 mg single-agent ieramilimab Q2W was comparable to the same dose in combination with 240 mg spartalizumab Q2W (Geo-mean AUC_tau_ (%CV): 477 day µg/mL (27.8%) vs 568 day* μg/mL (35.5%); Geo-mean C_max_ (%CV): 71.1 µg/mL (24.2%) vs 84.8 µg/mL (30.6%)). Exposure of 400 mg single-agent ieramilimab Q4W was similar to the same dose in combination with 400 mg spartalizumab Q4W (Geo-mean AUC_tau_ (%CV): 1220 day*μg/mL (36%) vs 1160 day* μg/mL (13.9%); Geo-mean C_max_ (%CV): 120 µg/mL (31.5%) vs 121 µg/mL (7.6%)).

The PK of spartalizumab in combination with different dose levels of ieramilimab were similar to the single-agent spartalizumab data at the same dose levels from a phase I study.^14^

### Efficacy

**Figure 4:**
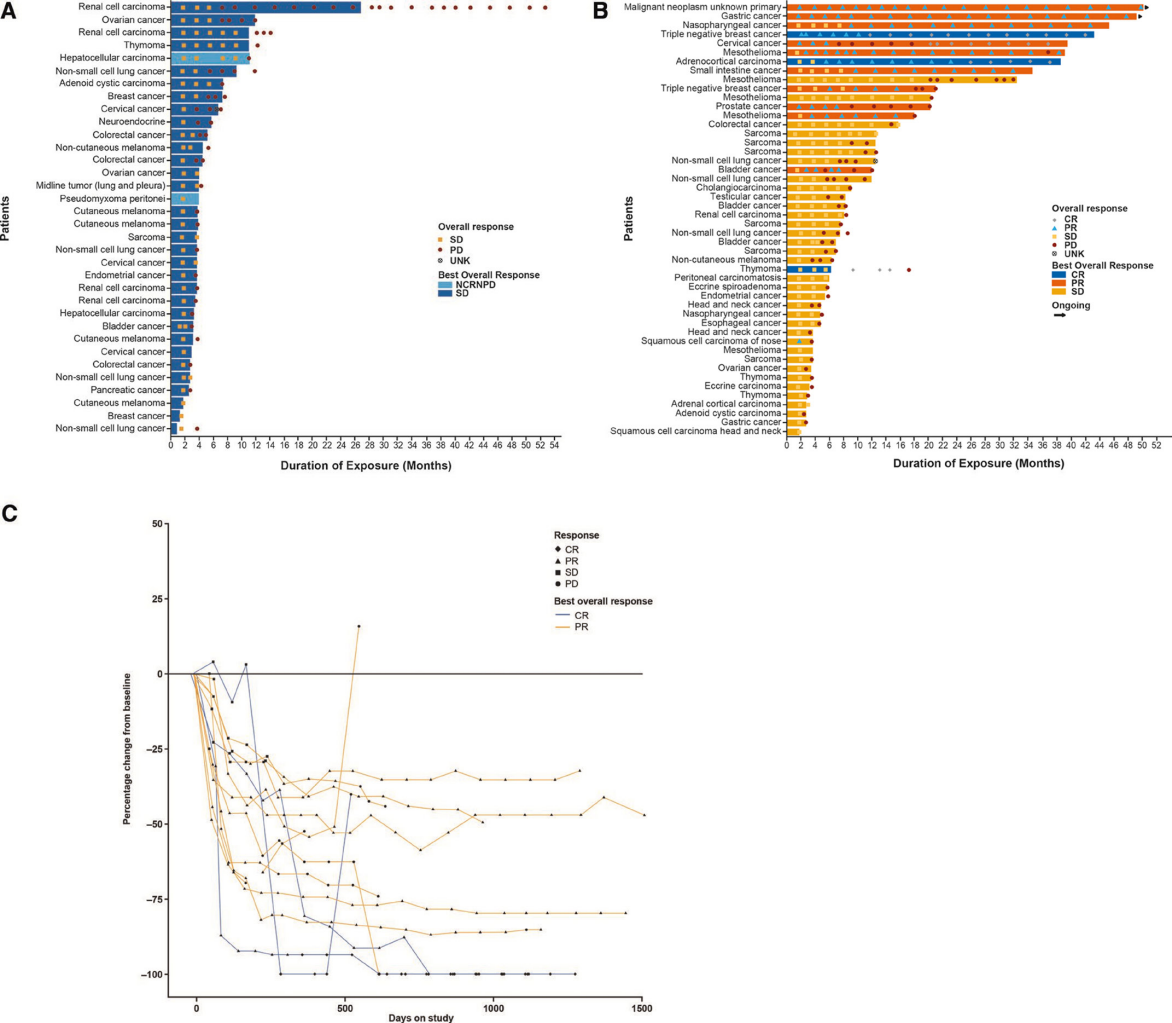
Duration of exposure and response plots. (A) Duration of exposure in patients receiving single-agent ieramilimab with best overall response of SD or NCRNPD, (B) Duration of exposure in patients receiving combination ieramilimab +spartalizumab with best overall response of CR, PR or SD, (C) Duration of response in patients receiving combination ieramilimab +spartalizumab with a best overall response of CR and PR. CR, complete response; NCRNPD, non-complete response/non-progressive disease (the presence of any non-target lesions or abnormal nodal lesions); PD, progressive disease; PR, partial response; SD, stable disease; UNK, unknown.

Median exposure was 8.07 weeks (range 2.0–116.4) and 12.57 weeks (range 2.0–218.0) in the single-agent and combination treatment group, respectively. In the single-agent cohort, 32 (23.9%) patients had stable disease (SD) as investigator-assessed, confirmed best overall response (BOR), 82 (61.2%) had PD, 2 (1.5%) had non-complete responses (CRs)/non-PD, and 18 (13.4%) had unknown responses (table 2), per RECIST V.1.1. Thirty-six patients (26.9%) had SD as investigator-assessed, confirmed BOR by irRC (online supplemental table A3, online only). Best percentage change of preselected target lesions from baseline is presented in online supplemental figure A3 (online only). Analysis of duration of exposure showed that six (4.5%) patients experienced SD for 6 months or longer (figure 4A).

**Table 2:** Investigator-assessed confirmed best overall response by Response Evaluation Criteria In Solid Tumors V.1.1

	All phase I SA patients (N=134) n (%)	All phase I combo patients (N=121) n (%)
Best overall response		
Complete response (CR)	0	3 (2.5)
Partial response (PR)	0	10 (8.3)
Stable disease (SD)	32 (23.9)	35 (28.9)
Progressive disease (PD)	82 (61.2)	55 (45.5)
Non-CR/non-PD (NCRNPD)	2 (1.5)	1 (0.8)
Unknown	18 (13.4)	17 (14.0)
Overall response rate (CR+PR) 90% CI	0 (0.0 to 2.2)	13 (10.7) (6.5 to 16.5)
Disease control rate (CR+PR+SD) 90% CI	34 (25.4) (19.3 to 32.3)	49 (40.5) (33.0 to 48.4)

CI, confidence interval; SA, single-agent.

In the combination group, 3 patients (2.5%) had CRs, 10 (8.3%) had partial responses (PRs), 35 (28.9%) showed SD, 55 (45.5%) had PD, 1 (0.8%) had a non-CR/non-PD, and 17 (14%) had unknown responses (table 2) per RECIST V.1.1. Of the 35 patients with SD as their BOR, 8 (23%) had received prior anti-PD-1 or anti-PD-L1 therapy. By irRC, 4 patients (3.3%) showed CRs, 11 patients had (9.1%) PRs, and 38 patients (31.4) showed SD (online supplemental table A3, online only). Responding patients had not received prior checkpoint blockade. Best percentage change of preselected target lesions from baseline is presented in figure 3C. Duration of exposure analysis revealed that eight patients (6.6%) experienced SD for 6 months or longer (figure 4B). The three patients who had CRs had thymoma, adrenocortical carcinoma, and triple-negative breast cancer (TNBC). The durations of responses in responding patients are shown in figure 4C. CT images of a patient with adrenocortical carcinoma who achieved a CR to treatment are shown in online supplemental figure A1 (online only). Resolution of TNBC skin metastases after eight cycles of treatment with ieramilimab in combination with spartalizumab is shown in online supplemental figure A2 (online only).

### Biomarkers

A total of 241/255 (94.5%) patients provided biopsy samples at screening, 10 of which were archival; 110/255 (43.1%) patients provided on-treatment biopsy samples during cycle 2 (n=35) or cycle 3 (n=74), and one was unscheduled.

**Figure 5:**
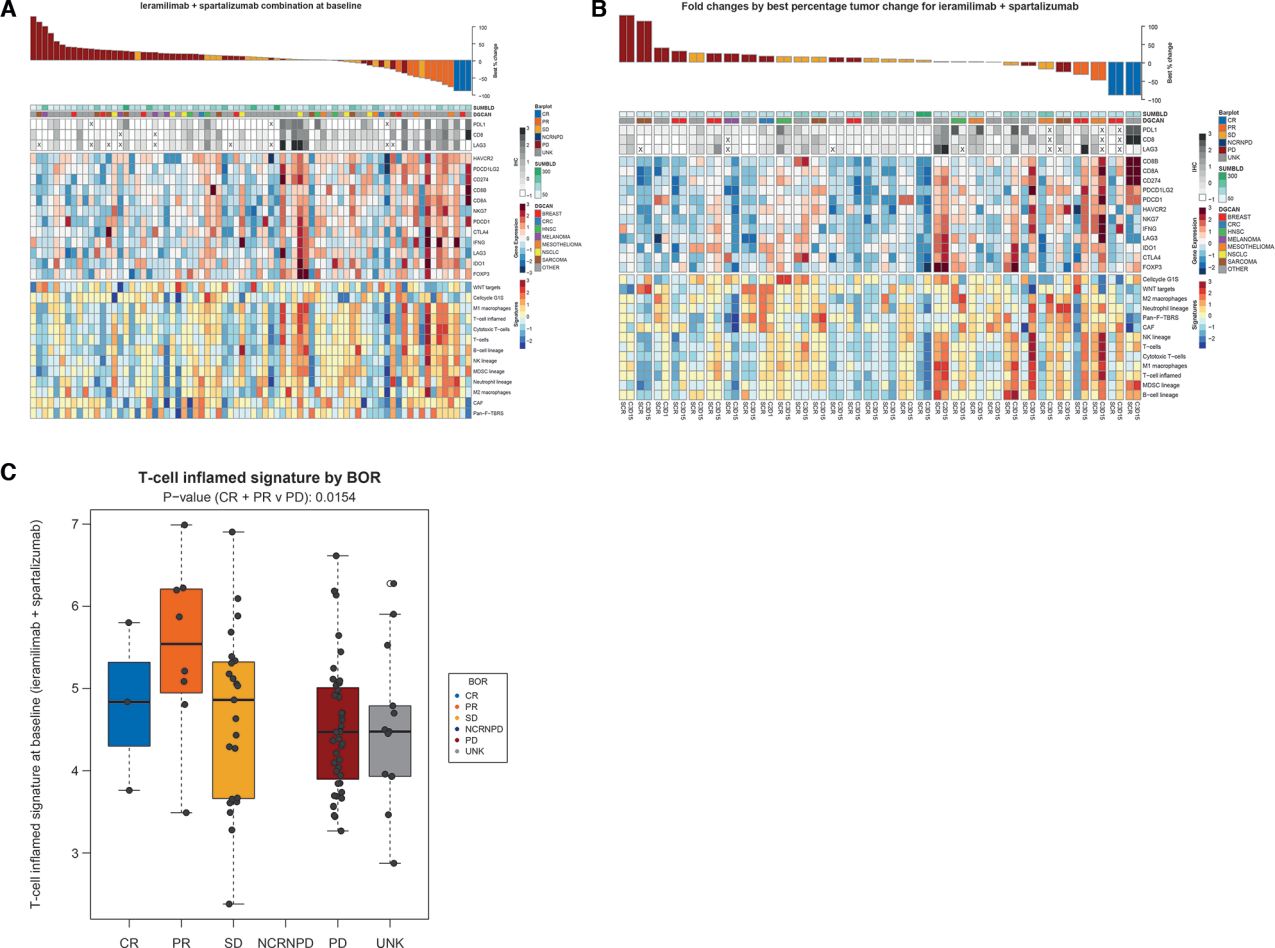
Effect of combination treatment (ieramilimab +spartalizumab) on immune-related markers. (A) IHC and RNA sequencing data at baseline (n=75), (B) IHC and RNA sequencing fold change data (n=28), (C) IFN-γ expression by BOR at baseline (n=91).: BOR, best overall response; CR, complete response; CRC, colorectal cancer; HNSC, head-neck squamous cell carcinoma; IFN, interferon-γ; IHC, immunohistochemistry; NCRNPD, non-complete response/non-progressive disease (the presence of any non-target lesions or abnormal nodal lesions); PD, progressive disease; PR, partial response; SD, stable disease; UNK, unknown.

IHC and RNA sequencing data of immune-related markers at baseline and fold changes for patients treated with a combination of ieramilimab and spartalizumab are shown in figure 5A, B. Overall, responding patients tended to have higher levels of immune gene expression at baseline (non-statistical trend). This was observed by IHC (CD8, LAG-3, and PD-L1), and similar trends were observed when investigating gene (*CD8A/B*, *LAG3*, indoleamine 2,3-dioxygenase, NK cell granule protein 7, *PDCD1* (PD-1), *CD274* (PD-L1), and *HAVCR2* (T-cell immunoglobulin and mucin domain-containing protein 3)) and gene signature (T cells, B cells, NK cells, and M1 macrophages) expression by RNA sequencing. Responding patients (CR, PR) in the combination treatment group showed a higher level of T-cell inflamed^22^ signature expression at baseline (p value (CR/PR vs PD): 0.0154) (figure 5C). T-cell inflamed signature results at baseline by BOR for the single-agent group are shown in online supplemental figure A4 (online only). Patients with tumors that exhibited stability or shrinkage tended to upregulate inflammatory gene expression signatures following ieramilimab and spartalizumab treatment, suggesting that this combination treatment may lead to enhanced T-cell activation within the tumor microenvironment (figure 5B).

## Discussion

Immune checkpoint blockade with anti-cytotoxic T-lymphocyte antigen 4 (CTLA-4) and/or anti-PD-(L)1 antibodies has transformed the treatment of several cancers, including melanoma and NSCLC, with improvements in overall survival.^23^ Many patients are, however, unresponsive to existing checkpoint inhibitors or develop resistance during treatment, underscoring the need for novel immunomodulatory approaches.^24^ Key immune-mediated mechanisms of resistance to checkpoint inhibitors include T-cell dysfunction, marked by the enhanced expression of co-inhibitory receptors; decreased T-cell priming and infiltration in the tumor microenvironment; suppression mediated by Tregs, myeloid-derived suppressor cells, and soluble factors; and loss of neoantigens/decreased antigen presentation. LAG-3 is an inhibitory receptor that is expressed in immune cells and has been shown, with PD-(L)1, to regulate T-cell exhaustion and inhibit an antitumor immune response.^5^ Compensatory upregulation of LAG-3 has been related to adaptive resistance to immune checkpoint blockade,^25^ supporting the hypothesis that targeting LAG-3 may be a promising therapeutic strategy to overcome immune checkpoint blockade resistance and improve patient outcomes.

This first-in-human, dose-escalation trial demonstrated that ieramilimab is well tolerated, both as a single agent and in combination with spartalizumab. Low-grade fatigue, gastrointestinal side effects, pruritus, and fever were among the most commonly occurring TRAEs associated with single-agent ieramilimab use. There was no increase in incidence of immune-mediated SAEs, consistent with the observation that LAG-3 deficiency alone does not result in autoimmunity in preclinical models.^26^ In contrast to combination checkpoint blockade with anti-CTLA-4 and anti-PD-1 agents, the immune-mediated toxicity of ieramilimab in combination with spartalizumab was comparable to that seen with spartalizumab alone.^14^ No new safety signals were identified compared with existing immune checkpoint inhibitor treatments.

Ieramilimab demonstrated approximately dose-proportional increases in exposure between the dose range of 1–15 mg/kg. Exposure of ieramilimab in combination with spartalizumab was within the range of exposure for both single-agent ieramilimab and spartalizumab, indicating no apparent drug–drug interaction between the two. Since there was no observed exposure response for safety or efficacy, and no MTD was reached, a target engagement receptor occupancy model was used to determine the RP2D, with the criteria of achieving 90% target engagement in >90% of patients. Similar approaches have been used to guide dosing of atezolizumab^27^ and sabatolimab.^28^

During dose escalation in a mixed population of advanced solid tumors, some of which had received prior treatment with checkpoint inhibitors, antitumor activity of single-agent ieramilimab was limited, consistent with preclinical models.^12^ In contrast, ieramilimab and spartalizumab combination treatment was associated with SD or tumor shrinkage, including three CRs by RECIST and an additional CR by irRC in a patient with cervical cancer (online supplemental table A3, online only). While most PRs occurred in patients with tumor types known to respond to anti-PD-1 antibodies, antitumor activity was observed in several tumor types where previous effectiveness of immunotherapy has not been established in a consistent way, including adrenocortical carcinoma and PD-L1-negative TNBC (online supplemental figure A1,2).^29^ In addition, the duration of response has exceeded 4 years in some patients, suggesting that long-term combination therapy is tolerable (figure 4B) and potentially augmented by LAG-3 blockade. For both ieramilimab doses at 400 mg Q3W and 800 mg Q4W, over 90% of patients were predicted to have at least 90% target engagement. At the alternative dosing regimen of ieramilimab 600 mg Q4W, 90% of patients were predicted to have at least 88% target engagement. This, therefore, indicates a comparable target engagement with ieramilimab doses at 600 mg Q4W and 800 mg Q4W.

In vitro, ieramilimab blocks the interaction between LAG-3 and both MHC-II and FGL-1, with high affinity. Elevated levels of FGL-1 in cancer may contribute to suppression of activated T cells and evasion of antitumor immunity,^8^ however, relative contributions of disrupting LAG-3 interactions with FGL-1 or MHC-II in patients is unclear. Although not addressed in this study, further translational investigation is warranted.

A large number of baseline tumor samples were collected to explore pharmacodynamic effects and potential efficacy predictors of ieramilimab, as both a single agent or in combination with spartalizumab. IHC and RNA sequencing analyses suggested that tumor stability or response following combination treatment was associated with baseline immune-inflamed gene expression patterns similar to the IFN-γ signature associated with response to the PD-1 inhibitor, pembrolizumab.^30^ In patients who received single-agent ieramilimab treatment, baseline T-cell inflamed signatures tended to be higher in tumor samples from those who exhibited SD (online supplemental figure A4C, online only). Among the heterogeneous tumors enrolled during the dose-escalation portion of the study, LAG-3 expression, per se, was not a predictive biomarker, except insofar as LAG-3 correlated with immune-inflamed gene expression patterns overall.

Consistent with the above observations regarding baseline immune gene expression, on-treatment biopsies suggested that patients with tumors that responded to ieramilimab in combination with spartalizumab demonstrated upregulation of already high baseline CD8 or T-cell inflamed expression levels. In several cases, however, tumor reduction occurred in the context of relatively immune-cold profiles at baseline, where on-treatment biopsies demonstrated increased levels of CD8 and PD-L1 following ieramilimab and spartalizumab treatment. The relative impact of ieramilimab on this effect is unknown and limited by the small number of PRs in this mixed group of tumor indications, as well as the smaller number of available on-treatment biopsies.

Despite preclinical models demonstrating synergistic antitumor activity with LAG-3 and PD-1 co-blockade,^12^ the modest antitumor activity observed in this clinical trial in a multitumor, unselected patient population, highlights the challenges in developing next-generation combination immunotherapies. Although the relative contribution of ieramilimab to antitumor efficacy could not be determined clinically or through translational analyses conducted in this study, a subset of patients experienced long-term clinical benefit with ieramilimab and spartalizumab. Consistent with a potential contribution of LAG-3 targeting, previous data on the combination of the LAG-3 inhibitor, relatlimab, with the PD-1 inhibitor, nivolumab, in patients with melanoma who had received prior immunotherapy, showed objective response rates of approximately 12%, with a disease control rate of 49% for the doublet.^31 32^ In the phase III RELATIVITY-047 study, relatlimab, in combination with nivolumab, demonstrated statistically significant progression-free survival benefit (10.1 months (95% CI: 6.4 to 15.7)) compared with nivolumab monotherapy (4.6 months (95% CI: 3.4 to 5.6), HR, 0.75 (95% CI: 0.6 to 0.9); p=0.0055) in patients with previously untreated metastatic or unresectable melanoma; this difference was likely driven by the LAG-3 positive (≥1%) subgroup.^33^ These results further highlight the clinical potential of dual LAG-3/PD-1 inhibition. Our phase I study showed responses to the dual anti-LAG-3/anti-PD-1 therapy in patients with various cancer indications, including confirmed CRs per RECIST V.1.1, in three patients with thymoma, adrenocortical carcinoma, and triple-negative breast cancer, as well as an additional CR by irRC in a patient with cervical cancer. Furthermore, the possible contribution of anti-LAG-3 to the durability of combination therapy response is supported by seven patients who received ieramilimab plus spartalizumab for over 3 years, including two of the patients achieving CR, one patient with a CR by irRC, plus four additional patients with mesothelioma, nasopharyngeal cancer, gastric cancer, and a malignant neoplasm of unknown primary who achieved PR. These data suggest that LAG-3 targeting may contribute to anti-PD-1 activity in different cancers beyond melanoma. Consistent with this, in the phase II part of our study, ieramilimab in combination with spartalizumab elicited durable responses not only in melanoma, but also in patients with mesothelioma and renal-cell carcinoma who had received prior treatment with anti-PD-(L)1 inhibitors.^34^ The clinical impact of targeting LAG-3 in combination with other immunotherapies warrants further investigation.

## Data Availability

Data are available upon reasonable request.
