# Supplementary material for: Phase I/II study of the LAG-3 inhibitor ieramilimab (LAG525) ± anti-PD-1 spartalizumab (PDR001) in patients with advanced malignancies
Source: J Immunother Cancer. 2022 Feb 25;10(2):e003776. doi: 10.1136/jitc-2021-003776 (PMC8883259; doi:10.1136/jitc-2021-003776)
Supplement: Supplementary data [file jitc-2021-003776supp001.pdf]

Supplemental Material

Supplemental figure A1 Computed tomography scans of a patient with adrenocortical carcinoma and multifocal lung metastasis at Baseline and cycle 38 following treatment with ieramilimab 240 mg + spartalizumab 400 mg Q4W

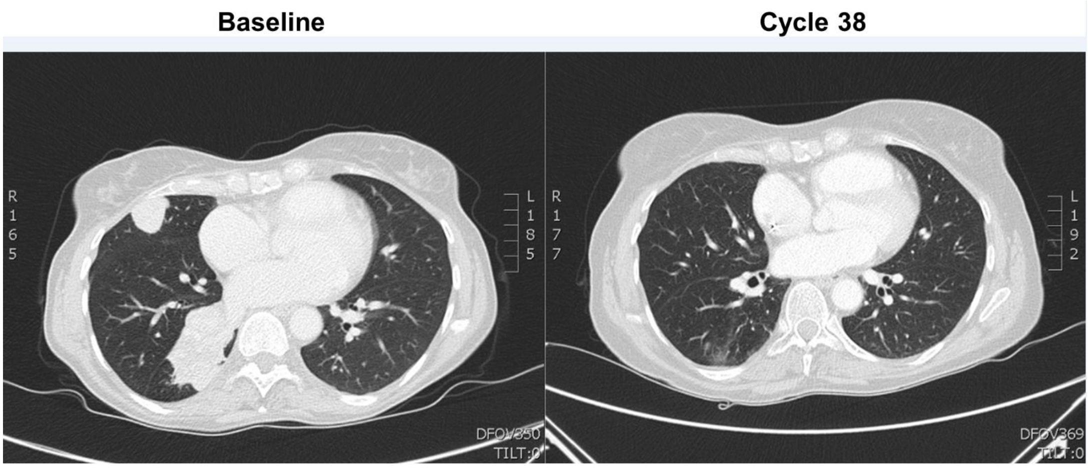

CT scans courtesy of Dr. P. Schöffski, University Hospitals Leuven, Leuven (Belgium).

The patient had a partial response on study day 169 followed by a complete response on study day 786.

Abbreviations: CT, computed tomography; Q4W, every 4 weeks.

Schöffski P, *et al. J Immunother Cancer* 2022; 10:e003776. doi: 10.1136/jitc-2021-003776

Supplemental figure A2 Resolution of skin metastases from PD-L1-negative TNBC after eight cycles of treatment with ieramilimab 240 mg + spartalizumab 300 mg, Q3W

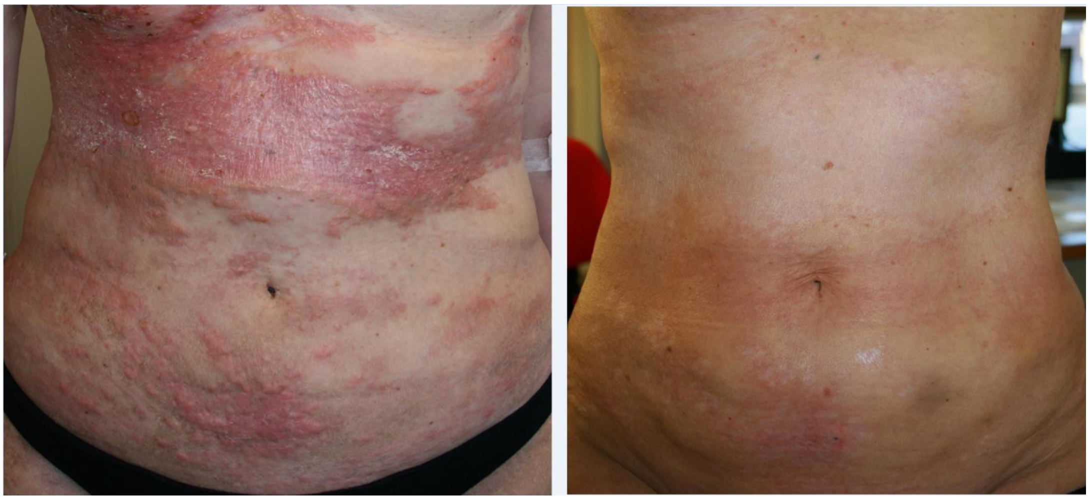

Images courtesy of Dr. Silvia Damian, Fondazione IRCCS Istituto Nazionale dei Tumori, Milan (Italy).

The patient provided a skin biopsy and had a responding tumor; they started treatment in 2016 and as of the data cutoff, they are still receiving treatment on the roll-over.

Abbreviations: Q3W, every 3 weeks; TNBC, triple-negative breast cancer.

Supplemental figure A3 Waterfall plot for best percentage change from baseline in sum of longest diameters based on local radiology review of RECIST 1.1 for patients treated with SA ieramilimab.

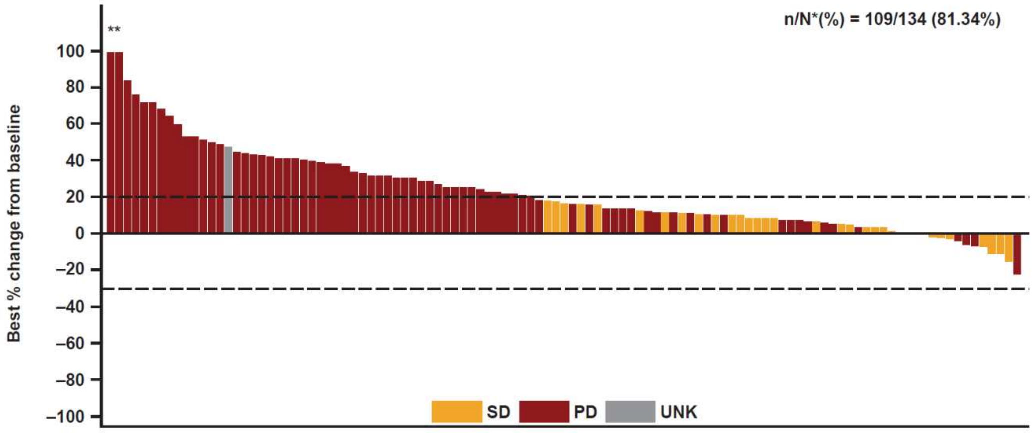

*Indicates the bars where best percentage change from baseline has been cut at 100%.

Abbreviations: CR, complete response; PD, progressive disease; PR, partial response; RECIST, Response Evaluation Criteria In Solid Tumors; SD, stable disease; UNK, unknown.

Schöffski P, *et al. J Immunother Cancer* 2022; 10:e003776. doi: 10.1136/jitc-2021-003776

Supplemental figure A4 Effect of single-agent ieramilimab treatment on immune-related markers. (A) IHC and RNA sequencing data at baseline (n=83), (B) IHC and RNA sequencing fold change data (n=37), (C) IFN-γ expression by BOR at baseline (n=105).

A

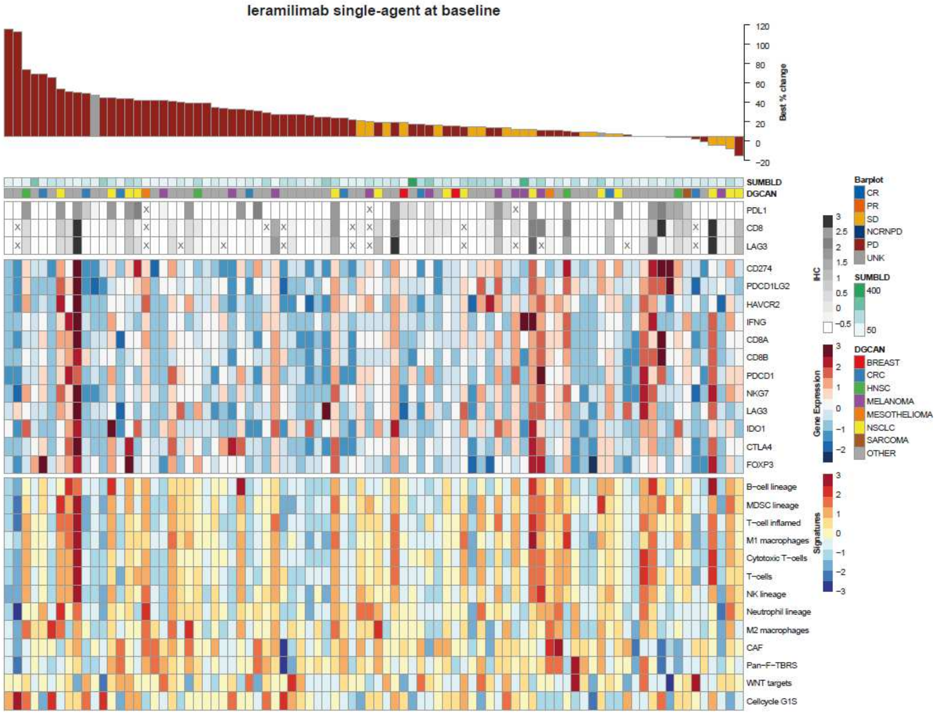

B

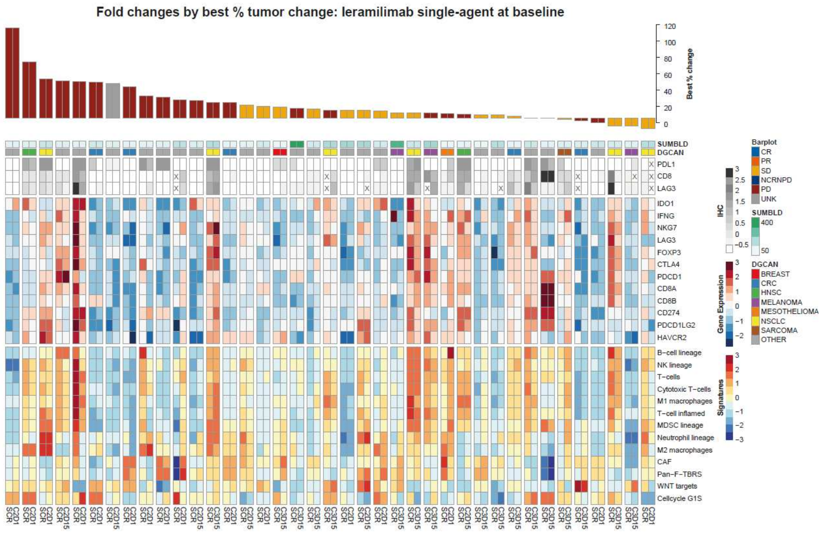

C

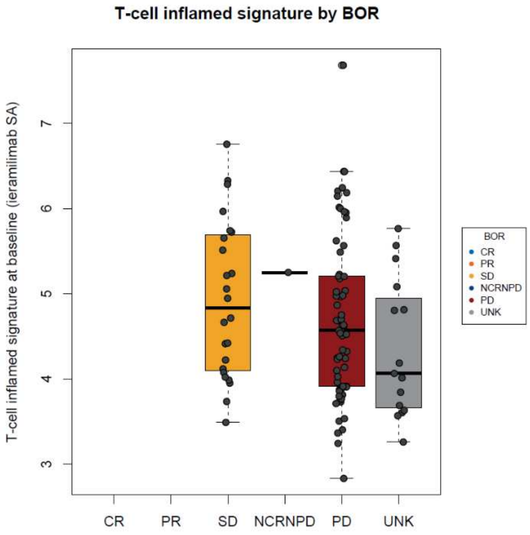

Abbreviations: BOR, best overall response; CAF, cancer-assisted fibroblast; CR, complete response; CRC, colorectal cancer; HNSC, head-neck squamous cell carcinoma; IFN-γ, interferon-gamma; IHC, immunohistochemistry; MDSC, myeloid-derived suppressor cell; NCRNPD, non-complete response/non-progressive disease (the presence of any non-target lesions or abnormal nodal lesions); NK, natural killer; NSCLC, non-small cell lung cancer; Pan-F-TBRS, pan-fibroblast TGFβ response signature; PD, progressive disease; PR, partial response; SA, single agent; SD, stable disease; UNK, unknown.

Supplemental table A1 Treatment-related AEs of any grade (≥2%).

	**All phase I SA patients (N=134) n (%)**		**All phase I combo patients (N=121) n (%)**		**All phase I patients (N=255) n (%)**	
	**All grades**	**Grade 3/4**	**All grades**	**Grade 3/4**	**All grades**	**Grade 3/4**
**Number of patients with at least one AE**	75 (56.0)	9 (6.7)	84 (69.4)	11 (9.1)	159 (62.4)	20 (7.8)
Fatigue	12 (9.0)	2 (1.5)	22 (18.2)	1 (0.8)	34 (13.3)	3 (1.2)
Nausea	11 (8.2)	2 (1.5)	15 (12.4)	0 (0.0)	26 (10.2)	2 (0.8)
Diarrhea	5 (3.7)	0 (0.0)	20 (16.5)	1 (0.8)	25 (9.8)	1 (0.4)
Pruritus	7 (5.2)	0 (0.0)	12 (9.9)	0 (0.0)	19 (7.5)	0 (0.0)
Vomiting	7 (5.2)	3 (2.2)	8 (6.6)	0 (0.0)	15 (5.9)	3 (1.2)
Decreased appetite	7 (5.2)	1 (0.7)	8 (6.6)	0 (0.0)	15 (5.9)	1 (0.4)
Rash	4 (3.0)	0 (0.0)	10 (8.3)	0 (0.0)	14 (5.5)	0 (0.0)
Hypothyroidism	5 (3.7)	0 (0.0)	8 (6.6)	0 (0.0)	13 (5.1)	0 (0.0)
Pyrexia	6 (4.5)	0 (0.0)	6 (5.0)	0 (0.0)	12 (4.7)	0 (0.0)
Myalgia	6 (4.5)	0 (0.0)	5 (4.1)	0 (0.0)	11 (4.3)	0 (0.0)
Rash maculo-papular	2 (1.5)	0 (0.0)	9 (7.4)	0 (0.0)	11 (4.3)	0 (0.0)
Asthenia	2 (1.5)	0 (0.0)	8 (6.6)	1 (0.8)	10 (3.9)	1 (0.4)
Arthralgia	4 (3.0)	0 (0.0)	5 (4.1)	0 (0.0)	9 (3.5)	0 (0.0)
Anemia	3 (2.2)	1 (0.7)	5 (4.1)	0 (0.0)	8 (3.1)	1 (0.4)
ALT increased	4 (3.0)	0 (0.0)	3 (2.5)	1 (0.8)	7 (2.7)	1 (0.4)
Dry mouth	4 (3.0)	0 (0.0)	3 (2.5)	0 (0.0)	7 (2.7)	0 (0.0)
AST increased	3 (2.2)	0 (0.0)	4 (3.3)	1 (0.8)	7 (2.7)	1 (0.4)
Amylase increased	4 (3.0)	2 (1.5)	2 (1.7)	0 (0.0)	6 (2.4)	2 (0.8)

Schöffski P, *et al. J Immunother Cancer* 2022; 10:e003776. doi: 10.1136/jitc-2021-003776

Abdominal pain	3 (2.2)	1 (0.7)	3 (2.5)	0 (0.0)	6 (2.4)	1 (0.4)
Stomatitis	1 (0.7)	0 (0.0)	5 (4.1)	0 (0.0)	6 (2.4)	0 (0.0)
Constipation	3 (2.2)	0 (0.0)	2 (1.7)	0 (0.0)	5 (2)	0 (0.0)
Lipase increased	3 (2.2)	2 (1.5)	2 (1.7)	2 (1.7)	5 (2)	4 (1.6)
Chills	1 (0.7)	0 (0.0)	4 (3.3)	0 (0.0)	5 (2)	0 (0.0)
Hypophosphatemia	1 (0.7)	0 (0.0)	4 (3.3)	1 (0.8)	5 (2)	1 (0.4)
Dysgeusia	2 (1.5)	0 (0.0)	3 (2.5)	0 (0.0)	5 (2)	0 (0.0)
Headache	2 (1.5)	0 (0.0)	3 (2.5)	0 (0.0)	5 (2)	0 (0.0)
Lethargy	4 (3.0)	0 (0.0)	0 (0.0)	0 (0.0)	4 (1.6)	0 (0.0)
Dry skin	3 (2.2)	0 (0.0)	1 (0.8)	0 (0.0)	4 (1.6)	0 (0.0)
Erythema	1 (0.7)	0 (0.0)	3 (2.5)	0 (0.0)	4 (1.6)	0 (0.0)
Muscular weakness	2 (1.5)	0 (0.0)	3 (2.5)	0 (0.0)	5 (1.6)	0 (0.0)
Blood creatinine increased	3 (2.2)	1 (0.7)	0 (0.00)	0 (0.0)	3 (1.2)	1 (0.4)
Malaise	3 (2.2)	0 (0.0)	0 (0.0)	0 (0.0)	3 (1.2)	0 (0.0)

Abbreviations: AE, adverse event; ALT, alanine aminotransferase; AST, aspartate aminotransferase; SA, single agent.

Supplemental table A2a Primary pharmacokinetic parameters for ieramilimab single agent.

Dosing regimen	Profiling Cycle	$AUC_{0\text{-}336h}$ Geo-mean (CV% geo-mean), day*µg/mL	AUC_{last} Geo-mean (CV% geo-mean), day*µg/mL	C_{max} Geo-mean (% CV geo-mean), µg/mL	Median T_{max} (range), h	$T_{1/2}$ eff Geo-mean (CV% geo-mean)
**Q2W ieramilimab**						
Ieramilimab 1 mg/kg (N=13)	Cycle 1	136 (36.9)	127 (41.5)	20.7 (34.6)	1.73 (1.5–2.45)	NA
	Cycle 3	273 (55.1)	235 (60.4)	32.9 (39.4)	1.83 (1.5–2.12)	11.8 (60.5)
Ieramilimab 3 mg/kg (N=12)	Cycle 1	495 (22.3)	495 (22.4)	71.1 (20.1)	1.6 (1.1–2.62)	NA
	Cycle 3	972 (34.4)	954 (34.1)	121 (24.9)	2.05 (1.55–2.08)	12.7 (34.3)
Ieramilimab 5 mg/kg (N=6)	Cycle 1	743 (22.0)	743 (21.9)	115 (22.6)	1.63 (1.17–2.13)	NA
	Cycle 3	2550 (NE)	2470 (NE)	272 (NE)	1.08 (1.08–1.08)	18.3 (NE)
Ieramilimab 10 mg/kg (N=6)	Cycle 1	1740 (38.7)	1730 (38.6)	239 (30.3)	1.63 (1.5–2.35)	NA
	Cycle 3	2340 (NE)	2330 (NE)	306 (NE)	1.55 (1.55–1.55)	9.2 (NE)
Ieramilimab 15 mg/kg (N=6)	Cycle 1	2740 (20.4)	2760 (21.4)	383 (26.5)	1.99 (1.48–2.07)	NA
	Cycle 3	4830 (25.3)	5150 (24.8)	600 (18.5)	1.67 (1.67–2.08)	12 (27.7)
Ieramilimab 240 mg (N=25)	Cycle 1	477 (27.8)	481 (28.7)	71.7 (24.2)	1.59 (1.5–2.17)	NA
	Cycle 3	845 (105.7)	357 (985.9)	108 (39.0)	1.58 (1.33–2.05)	13.3 (46.0)
Ieramilimab 400 mg (N=24)	Cycle 1	726 (31.7)	678 (32.2)	112 (27.5)	1.57 (1.47–2.45)	NA
	Cycle 3	1460 (45.7)	1070 (145.2)	158 (40.7)	1.5 (1.5–2.55)	12.8 (39.2)
	**Profiling day**	**Geo-mean $AUC_{0\text{-}672h}$ (CV% geo-**	**Geo-mean AUC_{last} (CV% geo-mean), day*µg/mL**	**Geo-mean C_{max} (% CV geo-mean), µg/mL**	**Median T_{max} (range), h**	**$T_{1/2}$ eff Geo-mean (CV% geo-mean)**

8

		mean), day*µg/mL				
**Q4W ieramilimab**						
Ieramilimab 3 mg/kg (N=5)	Cycle 1	682 (17.3)	610 (29.3)	65.2 (21.4)	1.58 (1.03–2.25)	NA
	Cycle 3	1120 (33.7)	845 (58.6)	77.4 (13.9)	2.08 (1.52–2.12)	16 (23.8)
Ieramilimab 5 mg/kg (N=6)	Cycle 1	1140 (45.6)	1120 (50.4)	116 (20.9)	2.1 (1.5–2.17)	NA
	Cycle 3	1480 (45.9)	1040 (77.1)	116 (22.8)	1.93 (1.57–2.3)	9.59 (23.8)
Ieramilimab 10 mg/kg (N=10)	Cycle 1	2550 (19.0)	2130 (31.6)	266 (13.8)	2.02 (1.5–2.28)	NA
	Cycle 3	3230 (NE)	804 (756.6)	222 (0.0)	1.83 (1.65–2)	NA
Ieramilimab 400 mg (N=5)	Cycle 1	1220 (36.0)	1220 (36.2)	120 (31.5)	1.5 (1.5–1.68)	NA
	Cycle 3	2380 (125.4)	1980 (180.8)	211 (100.7)	1.5 (1.5–1.5)	18.1 (86.8)

Supplemental table A2b Primary pharmacokinetic parameters for ieramilimab when in combination with spartalizumab.

	Profiling day	Geo-mean $AUC_{0\text{-}336h}$ (CV% geo-mean), day*µg/mL	Geo-mean AUC_{last} (CV% geo-mean), day*µg/mL	Geo-mean C_{max} (% CV geo-mean), µg/mL	Median T_{max} (range), h	$T_{1/2}$ eff Geo-mean (CV% geo-mean)
**Q2W ieramilimab + Q2W spartalizumab**						
Ieramilimab 0.3 mg/kg + spartalizumab 1 mg/kg (N=6)	Cycle 1	39.2 (29.6)	34.3 (34.6)	8.26 (31.5)	1.5 (1.5–1.65)	NA
	Cycle 3	52.6 (71.0)	51.9 (73.0)	8.5 (71.1)	1.72 (1.57–1.83)	7.2 (73.3)
Ieramilimab 1 mg/kg + spartalizumab 1 mg/kg (N=6)	Cycle 1	169 (19.6)	172 (20.6)	26.3 (14.0)	1.5 (0.933–1.53)	NA
	Cycle 3	248 (71.8)	249 (72.8)	30.5 (32.9)	1.51 (1.5–1.57)	11.6 (26.9)
Ieramilimab 80 mg + spartalizumab 80 mg (N=6)	Cycle 1	134 (32.2)	133 (31.9)	23.4 (27.3)	1.5 (1.47–1.52)	NA
	Cycle 3	249 (52.3)	242 (54.0)	34.7 (19.9)	1.5 (1.5–1.58)	13.4 (36.5)
Ieramilimab 80 mg + spartalizumab 240 mg (N=5)	Cycle 1	153 (36.5)	154 (35.8)	24.4 (29.0)	1.53 (1.5–1.58)	NA
	Cycle 3	282 (6.1)	213 (60.3)	33.2 (25.3)	1.5 (1.5–1.52)	12.5 (37.8)
Ieramilimab 240 mg + spartalizumab 240 mg	Cycle 1	568 (35.5)	560 (35.9)	84.8 (30.6)	1.55 (1.5–2.03)	NA
	Cycle 3	1930 (NE)	1050 (113.4)	137 (72.6)	1.53 (1.5–2)	17.3 (14.4)

(N=6)						
	**Profiling day**	**Geo-mean $AUC_{0\text{-}504h}$ (CV% geo-mean), day*µg/mL**	**Geo-mean AUC_{last} (CV% geo-mean), day*µg/mL**	**Geo-mean C_{max} (% CV geo-mean), µg/mL**	**Median T_{max} (range), h**	**$T_{1/2}$ eff Geo-mean (CV% geo-mean)**
**Q3W ieramilimab + Q3W spartalizumab**						
Ieramilimab 240 mg + spartalizumab 300 mg (N=18)	Cycle 1	598 (26.3)	586 (28.5)	71.7 (20.3)	1.52 (1.02–4.25)	NA
	Cycle 3	813 (46.4)	803 (45.9)	85.4 (28.8)	1.53 (1.48-3.77)	12.1 (31.2)
Ieramilimab 400 mg + spartalizumab 300 mg (N=5)	Cycle 1	887 (44.3)	887 (44.3)	118 (29.6)	1.5 (1.5–1.58)	NA
	Cycle 3	1190 (52.2)	1270 (61.0)	113 (17.6)	1.58 (1.58–1.58)	9.43 (9.3)
Ieramilimab 600 mg + spartalizumab 300 mg (N=11)	Cycle 1	1660 (27.9)	1570 (31.7)	211 (21.4)	1.57 (1.0–4.45)	NA
	Cycle 3	2400 (32.7)	1550 (117.3)	269 (21.2)	1.52 (1.0–1.55)	10.6 (42.2)
	**Profiling day**	**Geo-mean $AUC_{0\text{-}672h}$ (CV% geo-mean), day*µg/mL**	**Geo-mean AUC_{last} (CV% geo-mean), day*µg/mL**	**Geo-mean C_{max} (% CV geo-mean), µg/mL**	**Median T_{max} (range), h**	**$T_{1/2}$ eff Geo-mean (CV% geo-mean)**
**Q4W ieramilimab + Q4W spartalizumab**						
Ieramilimab 80 mg + spartalizumab 240 mg (N=7)	Cycle 1	192 (39.7)	182 (43.4)	26.8 (19.5)	1.52 (1.5–1.55)	NA
	Cycle 3	307 (160.9)	164 (305.2)	29.2 (54.5)	1.58 (1.5–1.75)	20.7 (NE)

Ieramilimab 400 mg + spartalizumab 400 mg (N=4)	Cycle 1	1160 (13.9)	1030 (17.3)	121 (7.6)	1.5 (1.5–1.5)	NA
	Cycle 3	2210 (4.6)	2240 (2.0)	174 (6.5)	1.54 (1.5–1.58)	23 (9.2)
Ieramilimab 800 mg + spartalizumab 400 mg (N=10)	Cycle 1	2940 (33.2)	2710 (42.0)	267 (30.8)	1.58 (1.5–2.07)	NA
	Cycle 3	3440 (50.3)	3580 (46.1)	334 (43.5)	1.58 (1.5–2.05)	16.8 (15.5)
Ieramilimab 1000 mg + spartalizumab 400 mg (N=6)	Cycle 1	3040 (29.0)	2890 (35.0)	291 (34.8)	1.58 (1.5–2.1)	NA
	Cycle 3	6790 (48.6)	6790 (48.7)	510 (14.3)	1.75 (1.5–2.0)	21.7 (74.3)

Abbreviations: AUC_{last}, area under the concentration-time curve from time 0 to last measurable concentration; C_{max}, maximum observed concentration; CV, coefficient of variation; D, day; n, number of patients with available pharmacokinetic parameter values; $T_{1/2}$, time taken for half the initial dose administered to be eliminated; T_{max}, time to maximum observed concentration.

Supplemental table A3 Investigator-assessed confirmed Best Overall Response by irRC.

	**All phase I SA patients (N=134) n (%)**	**All phase I combo patients (N=121) n (%)**
**Best overall response**		
Complete response (irCR)	0	4 (3.3)
Partial response (irPR)	0	11 (9.1)
Stable disease (irSD)	36 (26.9)	38 (31.4)
Progressive disease (irPD)	77 (57.5)	47 (38.8)
Non-irCR/non-irPD (NirCRNirPD)	1 (0.7)	1 (0.8)
Unknown (UNK)	20 (14.9)	20 (16.5)
Overall response rate (ORR: irCR+irPR)<br>90% CI	0<br>(0.0, 2.2)	15 (12.4)<br>(7.8, 18.4)
Disease control rate (DCR: irCR+irPR+irSD)<br>90% CI	37 (27.6)<br>(21.3, 34.7)	54 (44.6)<br>(36.9, 52.5)

Abbreviations: CI, confidence interval; ir, immune-related; irRC, immune-related response criteria.

Supplemental table A4 Proteins used for crystal structure determination.

Construct	Amino acid sequence in one letter code	SEQ ID NO
Human LAG-3 (P18627)	MWEAQFLGLLFLQPLWVAPVKPLQPGAE<u>VPVVWAQEGAPA</u><br><u>QLPCSPTIPLQDLSLLRRAGVTWQHQPDSGPPAAAPGHPLAP</u><br><u>GPHPAAPSSWGPRPRRYTVLSVGPGGLRSGRLPLQPRVQL</u><br><u>DERGRQRGDFSLWLRPARRADAGEYRAAVHLRDRALSCRL</u><br><u>RLRLG</u>QASMTASPPGSLRASDWVILNCSFSRPDRPASVHWF<br>RNRGQGRVPVRESPHHHLAESFLFLPQVSPMDSGPWGCILT<br>YRDGFNVSIMYNLTVLGLEPPTPLTVYAGAGSRVGLPCRLPA<br>GVGTRSFLTAKWTPPGGGPDLLVTGDNGDFTLRLEDVSQAQ<br>AGTYTCHIHLQEQQLNATVTLAIITVTPKSFGSPGSLGKLLCEV<br>TPVSGQERFVWSSLDTPSQRSFSGPWLEAQEAQLLSQPWQ<br>CQLYQGERLLGAAVYFTELSSPGAQRSGRAPGALPAGHLLLF<br>LILGVLSLLLLVTGAFGFHLWRRQWRPRRFSALEQGIHPPQA<br>QSKIEELEQEPEPEPEPEPEPEPEPEPEQL	1
Human LAG-3 expression construct	metdtlllwvlllwvpgstgVPVVWAQEGAPAQLPCSPTIPLQDLSLLR<br>RAGVTWQHQPDSGPPAAAPGHPLAPGPHPAAPSSWGPRPR<br>RYTVLSVGPGGLRSGRLPLQPRVQLDERGRQRGDFSLWLRP<br>ARRADAGEYRAAVHLRDRALSCRLRLRLGhhhhhh	2
LAG525 Fab heavy chain	QVQLVQSGAEVKKPGASVKVSCKASGFTLTNYGMNWVRQA<br>RGQRLEWIGWINTDTGEPTYADDFKGRFVFSLDTSVSTAYLQ<br>ISSLKAEDTAVYYCARNPPYYYGTNNAEAMDYWGQGTTVTV<br>SSASTKGPSVFPLAPCSRSTSESTAALGCLVKDYFPEPVTVS<br>WNSGALTSGVHTFPAVLQSSGLYSLSSVVTVPSSSLGTKTYT<br>CNVDHKPSNTKVDKRVESKYG	3

Schöffski P, *et al. J Immunother Cancer* 2022; 10:e003776. doi: 10.1136/jitc-2021-003776

LAG525 Fab light chain	DIQMTQSPSSLSASVGDRVTITCSSSQDISNYLNWYLQKPGQ SPQLLIYYTSTLHLGVPSRFSGSGSGTEFTLTISSLQPDDFATY YCQQYYNLPWTFGQGTKVEIKRTVAAPSVFIFPPSDEQLKSG TASVVCLLNNFYPREAKVQWKVDNALQSGNSQESVTEQDSK DSTYSLSSTLTLSKADYEKHKVYACEVTHQGLSSPVTKSFNR GEC	4

Supplemental table A5 Interactions between human LAG-3 and ieramilimab.

**LAG-3**		**Ieramilimab**		
**Amino acid**	**Number**	**Amino acid**	**Number**	**Chain**
Ser	45	Tyr	104	H
		Tyr	103	H
Pro	46	Tyr	103	H
		Tyr	102	H
		Tyr	104	H
Thr	47	Tyr	103	H
		Tyr	102	H
		Pro	101	H
Ile	48	Tyr	102	H
		Pro	101	H
Pro	49	Tyr	102	H
		Trp	50	H
		Pro	101	H
		Trp	50	H
		Asn	52	H
Leu	50	Tyr	102	H
		Pro	100	H
		Pro	101	H

Schöffski P, *et al. J Immunother Cancer* 2022; 10:e003776. doi: 10.1136/jitc-2021-003776

		Asn	99	H
		Tyr	102	H
		Glu	110	H
		Ala	111	H
		Pro	100	H
		Ala	109	H
		Trp	96	L
		Tyr	91	L
Gln	51	Trp	50	H
		Asn	35	H
		Trp	47	H
		Asn	99	H
		Trp	96	L
		Leu	94	L
Asp	52	Trp	50	H
Leu	53	Tyr	102	H
		Tyr	91	L
		Tyr	32	L
Ser	54	Tyr	92	L
		Asn	93	L
Leu	56	Tyr	102	H

Arg	57	Tyr	92	L
		Tyr	32	L
		Asn	93	L
Gln	128	Tyr	32	L
Arg (RGD motif)	129	Tyr	104	H
		Tyr	102	H
		Tyr	103	H
		Glu	110	H
		Tyr	32	L
		Tyr	50	L
		Tyr	91	L
Gly (RGD motif)	130	Tyr	104	H
		Tyr	102	H
Asp (RGD motif)	131	Tyr	104	H

Abbreviations: RGD, tripeptide Arg-Gly-Asp.

Schöffski P, *et al. J Immunother Cancer* 2022; 10:e003776. doi: 10.1136/jitc-2021-003776

Preclinical Methodology:

LAG-3 soluble neutralization Meso Scale Discovery (MSD) assay

Briefly, a 384-well standard binding MSD (Meso Scale Discovery) plate was coated with 2 µg/mL FGL-1-His (Sino Biological) and incubated overnight at 4°C. LAG-3-Fc-biotin (R&D; biotinylated in-house, final assay concentration of 200 pM) and titrations of ieramilimab IgG were co-incubated in a V-bottom 96-well polypropylene plate for 1 hour (h) at room temperature (RT). After blocking and washing, the LAG3-Fc-biotin/ieramilimab complex was added to MSD plates and incubated for 1 h at RT. Plates were washed, and 1 µg/mL streptavidin sulfotag (MSD) was added and incubated for 30 minutes at RT. After washing, MSD read buffer T was added, and plates were read plate on a Sector Imager 6,000 (MSD).

T follicular helper cell and B-cell co-culture

Peripheral blood mononuclear cells (PBMCs) were extracted from buffy coats from healthy human donors (Bern Blutspendezentrum) by isolation over Ficoll (Greiner Bio-One). After isolation, $CD19^{+}CD27^{-}IgM^{+}IgD^{+}$ naive B cells were isolated by positive selection according to manufacturer's protocol (Miltenyi Biotec) followed by fluorescence-activated cell sorting (FACS). $CD4^{+}$ T cells were then isolated from the negative fraction by negative selection according to manufacturer's protocol (Stemcell Technologies). Tfh cells ($CD4^{+}$CD45RA-$CXCR5^{+}$) cells were isolated by FACS on a FACS Aria III. T cell populations were then labelled with 2.5 mM CellTrace Violet (CTV; Thermofisher Scientific), and B cell populations with 0.5 mM Carboxyfluorescein succinimidyl ester (CFSE; Thermofisher Scientific). Labeled B and T cells were co-cultured at 60,000 cells/well total in a 96-well round bottom plate at a 1:1 ratio in RPMI medium (Thermofisher Scientific) and activated with 1 µg/mL Staphylococcal enterotoxin B (SEB Toxin Technology) for 5 days at 37°C, 5% CO_2 in the presence of 10 µg/mL ieramilimab or hIgG4 (S228P) isotype control. Supernatant was harvested at day 5, and IL-2, TNF-α, IL-10, and IFN-γ measured by MSD platform.

Ieramilimab/LAG-3 crystal structure

The crystal structure of a human LAG-3 (first IgV domain [D1], SEQ ID NO: 2, figure 1) bound to the Fab fragment of a humanized anti–LAG-3 antibody ieramilimab (SEQ ID NO: 3 and 4, Supplemental table A3, online only) was determined. As detailed below, LAG-3 was co-expressed with ieramilimab Fab in mammalian cells to produce purified complex. Protein crystallography was then employed to generate atomic resolution data for LAG-3 bound to ieramilimab Fab to define the epitope.

Protein production

The sequences of LAG-3 and ieramilimab Fab produced for crystallography are shown in Supplemental table 3 (online only). The construct of LAG-3 comprised residues 29 to 167 (underlined) of human LAG-3 (UniProt identifier P18627, SEQ ID NO: 1), along with N- and C-terminal residues from recombinant expression vector (shown in lower case letters, SEQ ID NO: 2). The N-terminal signal sequence from mouse IgG kappa light chain was used for secreted expression of LAG-3 and was cleaved during expression, leaving intact N-terminus of LAG-3. The C-terminus of LAG-3 was fused with a 6x His tag for purification. For ieramilimab Fab, the sequences of heavy (SEQ ID NO: 3) and light (SEQ ID NO: 4) chains are shown.

LAG-3 was co-expressed with ieramilimab Fab in Expi293 cells to produce complex for crystallography. In detail, 0.5 mg of plasmid encoding LAG-3 was mixed with 0.25 mg of plasmid encoding the heavy chain of ieramilimab Fab and 0.25 mg of plasmid encoding the light chain of ieramilimab Fab, diluted into 50 mL of Opti-MEM I medium (Life Technologies), and incubated with 2.5 mg of Polyethylenimine (Polysciences) in 50 mL of the same medium for 30 min. The mixture was then added into 1 L of Expi293 cells grown in suspension in Expi293 Expression medium (Life Technologies) at 2 million cells/mL at 37°C with 8% of CO_2 for transfection. After 5 days, the medium containing LAG-3/ ieramilimab Fab complex was harvested by centrifugation; 10 mL of Ni-NTA resin was added into the medium and

stirred at 4°C overnight. The following day the beads were packed into a gravity column and washed with 25 mM Hepes pH 7.4, 150 mM NaCl (HBS) supplemented with 20 mM of imidazole. The complex was eluted with three column volumes (CV) of HBS with 500 mM of imidazole, and dialyzed in HBS at 4°C overnight. The next day, the complex was incubated with 1/10 (w/w) of PNGaseF (purified in-house) at 37°C overnight to remove N-linked glycosylation. After deglycosylation, the mixture was bound back to 5 mL of Ni-NTA resin, washed with HBS to remove PNGaseF and eluted with HBS plus 500 mM of imidazole. The eluent was then concentrated and loaded onto HiLoad 16/600 Superdex 75 PG (GE Healthcare) size exclusion column equilibrated in HBS. Peak fractions containing purified LAG-3/ ieramilimab Fab complex were analyzed by SDS-PAGE, pooled and concentrated for crystallization.

Crystallization and structure determination

LAG-3/ieramilimab Fab complex was concentrated to 20 mg/mL, centrifuged at 20,000 g for 10 min, and screened for crystallization. Crystals for data collection were grown by hanging drop vapor diffusion at 20°C. In detail, 0.1 µL of the LAG-3/ ieramilimab Fab complex was mixed with 0.1 µL of reservoir solution containing 0.2 M tri-potassium citrate monohydrate pH 8.3 and 20% (w/v) PEG3350. The drop was then equilibrated against 45 µl of the same reservoir solution. Before data collection, the crystals were shortly soaked in cryo-protectant (80% [v/v] reservoir solution plus 20% [v/v] ethylene glycol) and flash cooled in liquid nitrogen.

Diffraction data were collected at beamline 17-ID at the Advanced Photon Source (Argonne National Laboratory, USA), and processed using Autoproc (version 1.1.5, Global Phasing, LTD). The data of LAG-3/ieramilimab Fab were processed to 2.7 Å in space group C2 with cell dimensions a=195.0 Å, b=68.5 Å, c=115.0 Å, alpha=90°, beta=122.9°, and gamma=90°. The structure of the complex was solved by molecular replacement using Phaser (version 2.5.5, McCoy *et al.*, (2007) J. Appl. Cryst. 40:658-674) with an in-house Fab structure as search model. The structure of LAG-3 D1 was built from scratch using Buccaneer (K.

Cowtan (2006) Acta Cryst. D62:1002-1011) in the CCP4 program suite (Winn *et al.*, (2011) Acta. Cryst. D67:235-242). The final model was built in COOT (version 0.6 pre, Emsley & Cowtan (2004) Acta Cryst. D60:2126-2132) and refined using Phenix (version 1.9, Afonine *et al.*, (2012) Acta Cryst. D68:352-367). The R_{work} and R_{free} values were 18.2 % and 24.2 %, respectively; and the root-mean-square (r.m.s) deviation values of bond lengths and bond angles were 0.009 Å and 1.4 °, respectively.

Epitope was defined as residues of LAG-3 that contain atoms within 5 Å to any atom in ieramilimab Fab, identified by CONTACT in CCP4 program suite (version 6.2.0, Winn *et al.*, (2011) Acta. Cryst. D67:235-242) and listed in Supplemental table 4 (online only). There were two copies of LAG-3/ ieramilimab Fab complexes in the asymmetric unit (the smallest unique unit in the crystal), only those antibody-contacting residues that were common in both copies are listed as epitope residues.

Epitope of ieramilimab on LAG-3

The crystal structure of the LAG-3/ ieramilimab Fab complex was used to identify the epitope of ieramilimab on LAG-3. The interaction surface on LAG-3 by ieramilimab was formed by several continuous and discontinuous (*i.e.* noncontiguous) sequences: namely residues Ser45, Pro46, Thr47, Ile48, Pro49, Leu50, Gln51, Asp52, Leu53, Ser54, Leu56, Arg57 (these 12 residues are from BC loop), Gln128, Arg129, Gly130, and Asp131 (these four residues are from DE loop), as detailed in Supplemental table A4 (online only). These residues form the three-dimensional conformational epitope that is recognized by ieramilimab (figure 1A, B). Notably LAG-3 has a unique structure called “extra loop” (figure 1A) in between strands C and C’ of D1, which is absent in other homologous molecules like CD4, but is far distal to the ieramilimab epitope.

Supplemental table A5 (online only) elaborates interactions between human LAG-3 and ieramilimab. LAG-3 residues are numbered as in UniProt entry P18627 (SEQ ID NO: 1; Supplemental table A3, online only). The antibody residues are numbered based upon their linear amino acid sequence (SEQ ID NO: 3 and 4) and corresponding chains are labeled

(“H” for heavy chain, “L” for light chain). LAG-3 residues shown here have at least one atom within 5 Å to an atom in ieramilimab, to account for potential water mediated interactions.

RNA extraction

Sections of 5-µm (±1 µm) thickness were cut from all blocks received. A pathologist visually inspected archival formalin-fixed paraffin-embedded (FFPE) slides and freshly cut slides from the tumor blocks to identify and notate the approximate percentage of tumor content in the region of interest (ROI) and total tumor area (mm^2). Depending on the tumor cell content, four to 12 slides were macro dissected and used for DNA/RNA isolation. If the ROI contained <10% tumor content, further processing was canceled. DNA/RNA was coextracted from all samples available using the Qiagen AllPrep RNA/DNA Extraction from FFPE Tissue Kit.

RNA sequencing

Ribosomal RNA (rRNA) from extracted total RNA was depleted using RNAseH. The rRNA-depleted sample was then fragmented, converted to cDNA, and carried through the remaining steps of next-generation sequencing library construction: end repair, A-tailing, indexed adaptor ligation, and PCR amplification using the TruSeq RNA v2 Library Preparation kit (Illumina). The captured library was pooled with other libraries, each having a unique adaptor index sequence, and applied to a sequencing flow cell. The flow cell underwent cluster amplification and massively parallel sequencing by synthesis using Illumina v4 chemistry and paired-end 100 bp reads (Illumina).

Sequence data were aligned to the reference human genome (build hg19) using STAR.[1] Mapped reads were then used to quantify transcripts with HTSeq[2] and the Refseq GRCh38 v82 gene annotation. Gene expression data were normalized using trimmed mean of M-value normalization as implemented in the edgeR R/Bioconductor package.[3] Hierarchical clustering was performed using Euclidean distance for samples and Pearson correlation for

genes and gene sets; features were also ordered using the optimal leaf ordering algorithm as implemented in the R package cba. Pathway/gene set expression was derived using the geometric mean expression of all genes in each set. For pathway analyses, we used 1329 gene sets from MSigDB v6.2 C2 Canonical Pathways[4–6] and added in-house and published gene sets.[7] Pathways were ranked in unbiased analyses using two-sided Wilcoxon rank-sum tests. All analyses were performed in R v.3.6.1.

Guidelines for immune-related response criteria (irRC)

The currently used irRC uses unidimensional measurements to assess tumor response. The primary difference between irRC and RECIST 1.1 is the definition of progressive disease. The definitions of baseline target/non target lesions, number of lesions selected at baseline, the criteria for lesion measurement method of evaluation of response and definition of response are the same for irRC and RECIST 1.1

New lesions and non-target lesions: in irRC a new lesion does not automatically indicate progressive disease.

New measurable lesions were defined using the same criteria as for baseline target lesions in RECIST v1.1. New measurable lesions were prioritized according to size, and the largest lesions were selected as new measured lesions. Up to five new measurable lesions (and a maximum of two per organ) were allowed in total and were included in the overall tumor assessment.

Non-target lesions (baseline and new non-measurable lesions) were used primarily for determination of complete response (CR). The RECIST v1.1 definition for the assessment of non-target lesions was applied. A CR required that all non-target lesions disappeared (both those present at baseline and any new non-measurable lesions that appeared during the study). If after worsening, a non-target lesion became measurable, it was followed as a non-

Schöffski P, *et al. J Immunother Cancer* 2022; 10:e003776. doi: 10.1136/jitc-2021-003776

target lesion. Worsening of non-target lesions and new non-measurable lesions only indicated disease progression if there was unequivocal evidence of disease progression.

Supplemental References

1. Dobin A, Davis CA, Schlesinger F, *et al*: STAR: ultrafast universal RNA-seq aligner. *Bioinformatics* 2013;29:15–21.
2. Anders S, Pyl PT, Huber, W. HTSeq--a Python framework to work with high-throughput sequencing data. *Bioinformatics* 2015;31:166–169.
3. Robinson MD, McCarthy DJ, & Smyth GK. edgeR: a Bioconductor package for differential expression analysis of digital gene expression data. *Bioinformatics* 2010;26:139–140.
4. Subramanian A, Tamayo P, Mootha VK, *et al*: Gene set enrichment analysis: a knowledge-based approach for interpreting genome-wide expression profiles. *Proc Natl Acad Sci USA* 20015;102:15545–15550
5. Liberzon A, Birger C, Thorvaldsdóttir H, *et al*: The Molecular Signatures Database (MSigDB) hallmark gene set collection. *Cell Syst* 2015;1:417–425.
6. Liberzon A, Subramanian A, Pinchback R, *et al*: Molecular signatures database (MSigDB) 3.0. Bioinformatics 2011;27:1739–1740.
7. Dummer R, Lebbé C, Atkinson V, *et al*: Combined PD-1, BRAF and MEK inhibition in advanced BRAF-mutant melanoma: safety run-in and biomarker cohorts of COMBI-i. *Nature Medicine* 2020;26:1557–1563.

Schöffski P, *et al*. *J Immunother Cancer* 2022; 10:e003776. doi: 10.1136/jitc-2021-003776
